# PSMA‐Targeting Macrophage Membrane‐Coated Nanoparticles for Precision Diagnosis and Combination Therapy of Prostate Cancer

**DOI:** 10.1002/EXP.20240393

**Published:** 2026-02-22

**Authors:** Keying Zhang, Bo Gao, Jingwei Wang, Yu Li, Chao Xu, Fa Yang, Shaojie Liu, Hongji Li, Chao Zhang, Xiangliang Meng, Zhaokun Shi, Rui Zhang, Ruili Zhang, Zhongliang Wang, Weihong Wen, Qiang Zhang, Yuankang Zou, Donghui Han, Huijie Bian, Weijun Qin

**Affiliations:** ^1^ Department of Urology Xijing Hospital Fourth Military Medical University Xi'an China; ^2^ Institute of Orthopaedic Surgery Xijing Hospital Fourth Military Medical University Xi'an China; ^3^ The First Affiliated Hospital Southern University of Science and Technology Shenzhen China; ^4^ Department of Urology Daping Hospital Army Medical University Chongqing China; ^5^ State Key Laboratory of Cancer Biology Department of Immunology Fourth Military Medical University Xi'an China; ^6^ Engineering Research Center of Molecular & Neuroimaging Ministry of Education School of Life Science and Technology Xidian University Xi'an China; ^7^ Institute of Medical Research Northwestern Polytechnical University Xi'an China; ^8^ Department of Medicine Division of Hematology/Oncology Northwestern University Feinberg School of Medicine Chicago, IL USA; ^9^ Department of Occupational and Environmental Health and the Ministry of Education Key Lab of Hazard Assessment and Control in Special Operational Environment School of Public Health Fourth Military Medical University Xi'an China; ^10^ National Translational Science Center for Molecular Medicine & Department of Cell Biology Fourth Military Medical University Xi'an China

**Keywords:** combined treatment, immunomodulation, macrophage membrane‐coated nanoparticle, prostate cancer, prostate‐specific membrane antigen, targeted multimodal imaging

## Abstract

Prostate cancer (PCa) is the most frequently diagnosed cancer in males. Advanced PCa is invasive and may spread rapidly. Current strategies could not fulfill the requirement for clinical application; thus, novel therapeutic strategies are still urgently needed. Nanoparticles are a promising strategy for targeted drug delivery and cancer treatment; however, the strong exogeneity and weak targeting limit their further application. Here, we developed a novel macrophage membrane‐coated nanoparticle that has transmembrane‐expressed gy‐1, a single‐chain antibody fragment (scFv) against prostate‐specific membrane antigen (PSMA), to endow the immune evasion and targeting property, which we named P‐MMCNPs. The Fe_3_O_4_@Au nanoparticles were used as the core of P‐MMCNPs, which confer P‐MMCNPs with the properties of multimodal imaging and photothermal therapy (PTT). Anti‐tumor cytotoxic drug maytansine (DM1) was loaded into the nanoparticles to obtain cytotoxicity. P‐MMCNPs were shown to have immune evasion capacity and prolonged circulation time and can be specifically distributed in PSMA‐positive tumors, thus enabling targeted imaging and targeted drug delivery. The macrophage membrane‐coated nanoparticles combined to inhibit tumor growth in vivo when loaded with DM1 and treated with PTT. Additionally, we found that P‐MMCNPs alone could inhibit tumor growth, which may be caused by cytokine neutralization by the macrophage membrane. Our work demonstrates that the innovative P‐MMCNPs serve as a versatile platform. This platform improves PCa‐targeted diagnostic and therapeutic efficacy while avoiding side effects. Moreover, it holds promise for expanding into the diagnosis and treatment of other diseases, with potential for clinical translation. Additionally, it offers novel insights into nanomedicine‐based combination therapy.

## Graphical abstract

Advanced prostate cancer is highly invasive and prone to rapid metastasis, creating an urgent need for novel therapeutic strategies. We developed a PSMA‐targeting macrophage membrane‐coated nanomedicine (P‐MMCNPs@DM1). Leveraging the immune‐evasive and active‐targeting properties of macrophage membrane coating, P‐MMCNPs@DM1 effectively managed both primary and metastatic prostate tumors through combined anti‐tumor effects.

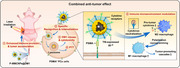

## 1 Introduction

Prostate cancer (PCa) is the most frequently diagnosed malignancy in men. Although androgen deprivation therapy and chemotherapy are the main treatments for advanced PCa, disease progression to androgen independence and the emergence of chemoresistance often occur within two years, resulting in a poor 5‐year survival rate of ∼30% [1, 2]. These limitations underscore the urgent need for novel targeted therapies.

The Fe_3_O_4_@Au nanoparticles (FeAuNPs) are well‐known for their high drug‐loading capacity, tunable photothermal properties, and intrinsic MRI/CT imaging capabilities, making them highly promising for tumor diagnosis and therapy [3]. They are particularly suitable for combination therapies, as photothermal therapy (PTT) offers rapid but transient tumor ablation, while chemotherapy provides a slower yet sustained effect; FeAuNPs enable the integration of both modalities to enhance therapeutic efficacy. However, their strong exogeneity and poor targeting often lead to rapid clearance by the immune system and off‐target accumulation [4, 5]. To overcome these limitations, various surface engineering strategies have been developed, among which cell membrane coating stands out for imparting biological functionalities such as enhanced biocompatibility, prolonged circulation, immune evasion, and homotypic targeting [6]. Membranes derived from erythrocytes [7], immune cells [8], platelets [9], cancer cells [10], and stem cells [11] have been explored, with erythrocyte‐derived nanosponges and platelet membrane‐coated nanomedicines already progressing into clinical Ib/IIa trials [9, 12]. Macrophage membranes are another attractive option due to their immune evasive properties, tumor‐homing tendencies, and immunomodulatory potential [13]; however, their homing effect is primarily mediated by chemokines and adhesion molecules, which lack specific cellular recognition and thereby limit delivery precision [6, 14, 15].

Prostate‐specific membrane antigen (PSMA), a type II transmembrane protein, is highly overexpressed in poorly differentiated, metastatic, and hormone‐refractory PCa, making it an ideal molecular target [16]. In our previous study, we identified a PSMA‐specific single‐chain antibody fragment (scFv), termed gy1, from a yeast‐display naïve human scFv library. The gy1 binds specifically to the extracellular domain of PSMA and mediates efficient internalization into PSMA‐positive cells [17, 18, 19]. We further developed a full‐length human antibody (PSMAb) based on gy1 and demonstrated its efficacy in siRNA‐targeted delivery for PCa therapy [17, 18, 19].

**FIGURE 1:**
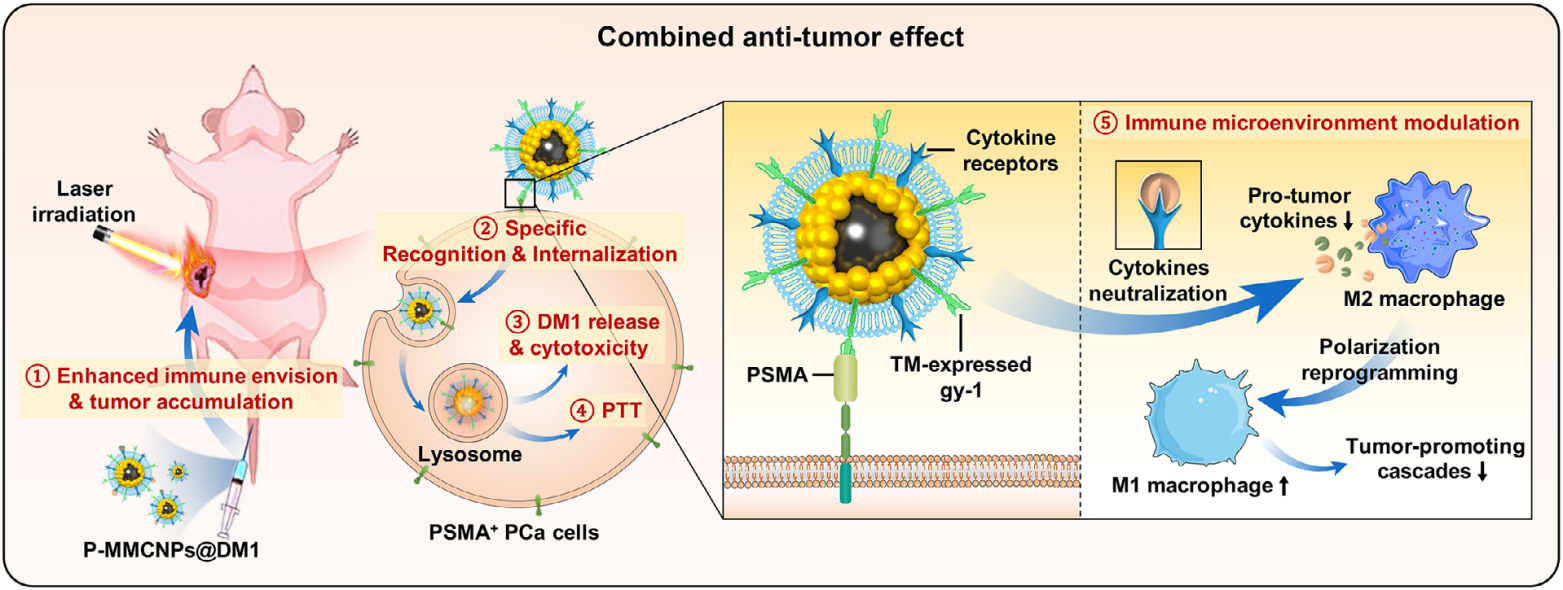
Schematic diagram to show PCa‐active targeting and combined anti‐tumor effects of P‐MMCNPs@DM1 after intravenous injection. P‐MMCNPs have a core‐shell structure, with a macrophage membrane that has TM‐expressed gy1‐enhanced green fluorescent protein (EGFP) fusion protein as the shell and FeAuNPs as the core. Then, the cytotoxic drug DM1 was loaded to obtain the P‐MMCNPs@DM1. When P‐MMCNPs@DM1 was injected into tumor‐bearing mice, the TM‐expressed gy1 could specifically recognize, bind with, and internalize into PSMA^+^ PCa cells, in which DM1 was released from the lysosome to induce cytotoxicity. Together with PTT treatment, it could result in a combined anti‐tumor effect. In addition, the macrophage membranes of P‐MMCNPs also neutralize pro‐tumor cytokines and block the M2 macrophage‐mediated tumor‐promoting cascades.

Here, we designed a novel kind of macrophage membrane‐coated nanoparticle named P‐MMCNPs@DM1, which was composed of two parts. The outer part was the macrophage membrane, which stably transmembrane (TM)‐expressed PSMA scFv (gy1) to ensure the targeting capacity and immune escape, and the inner part was FeAuNPs, coupled with the microtubular inhibitor maytansine (DM1). Cell‐specific recognition and internalization and specific distribution of P‐MMCNPs were examined in vitro and in vivo. This dual‐component design not only retains the prolonged circulation capability conferred by macrophage membrane camouflaging but, more importantly, integrates PSMA‐targeting ligands to achieve enhanced tumor‐homing specificity and cellular internalization. These synergistic features collectively contribute to improved tumor accumulation and drug delivery efficiency, thereby potentially amplifying the therapeutic efficacy of the nanoplatform in PCa treatment. The combined anti‐tumor effect induced by FeAuNPs‐related PTT and DM1‐related cytotoxicity was examined. We found that P‐MMCNPs@DM1 exhibited impressive PSMA targetability and a combined anti‐tumor effect for the successful management of primary and metastatic prostate tumors and concomitant damages. In addition, we found that P‐MMCNPs alone could also inhibit tumor growth, which is caused through cytokine neutralization by the macrophage membrane. Our work demonstrates that the novel P‐MMCNPs are a versatile platform for the delivery of cytotoxic drugs to enhance the therapeutic efficacy of PCa (Figure 1).

## 2 Results and Discussion

### 2.1 Preparation and Characterization of P‐MMCNPs

**FIGURE 2:**
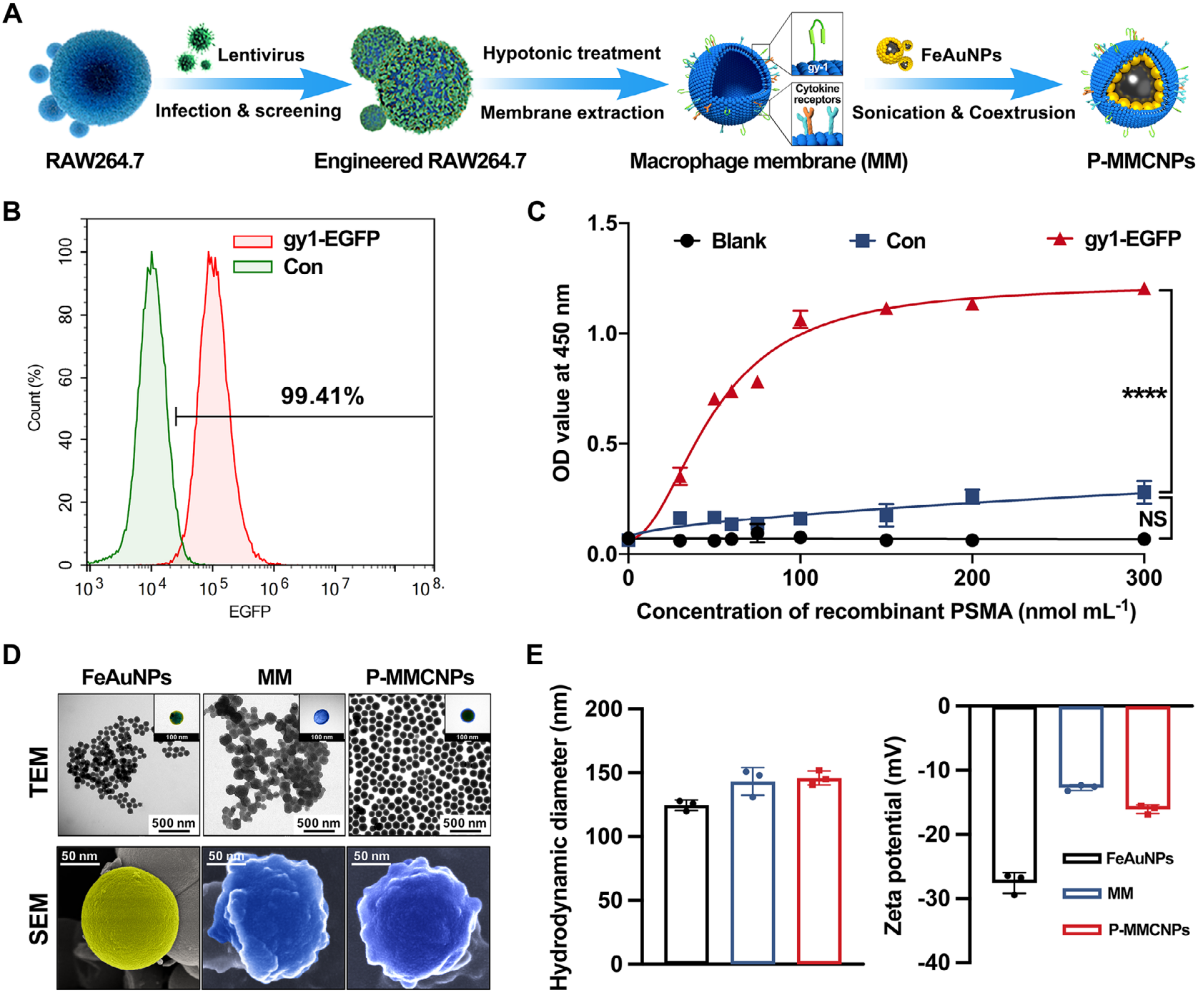
Preparation and characterization of P‐MMCNPs. (A) Schematic diagram to show the design and fabrication of P‐MMCNPs. Anti‐PSMA scFv (gy1)‐EGFP fusion protein was stably TM‐expressed in RAW264.7 cells, and the cell membrane was extracted and assembled with FeAuNPs to obtain P‐MMCNPs. MM represents Raw264.7^gy‐1‐EGFP^ macrophage membrane, (B) Flow cytometry to show the expression of gy1‐EGFP fusion protein on Raw264.7^gy1‐EGFP^ cells, (C) Cellular ELISA to analyze the binding affinity of Raw264.7^gy1‐EGFP^ to PSMA. Data were analyzed using one‐way ANOVA, (D) TEM and SEM images of FeAuNPs, MM, and P‐MMCNPs. Pseudo‐color processing was employed to highlight the structural features. Scale bars, 500 nm, 100 nm, and 50 nm, and (E) Hydrodynamic diameter and zeta potential of FeAuNPs, MM, and P‐MMCNPs. Data were shown as mean ± SD from three independent experiments. ^****^ *P* < 0.0001; **Abbreviation**: NS, no significant.

Bioinformatics analysis showed that PSMA exhibits prostate‐specific expression that intensifies with the progression of PCa malignancy, underscoring its potential as a target for both diagnosis and therapy of PCa (Supplementary Figure S1). Leveraging the advantages of macrophage membrane coating, we designed novel membrane‐coated nanoparticles by using the membranes of macrophages that have TM‐expressed PSMA scFv (named gy1, Figure 2A), aiming to overcome the shortcomings of strong exogeneity and weak targeting in nanoparticles. To obtain the macrophage membrane, Raw264.7 cells were infected with gy1‐overexpressing lentivirus, which contains the fusion gene of gy1, the transmembrane and hinge region of CD8, and intracellular EGFP (named Raw264.7^gy‐1‐EGFP^). As shown in Figure 2B and Supplementary Figure S2, high‐level (near 100%) expression of the gy‐1‐EGFP fusion protein or EGFP fluorescent labeling was easily detected by confocal laser scanning microscopy (CLSM) and flow cytometry. Furthermore, utilizing Western blotting, we verified the successful expression of the gy‐1‐EGFP fusion protein in Raw246.7^gy‐1‐EGFP^ cells, with the observed molecular weight closely aligning with the predicted 64 kDa (Supplementary Figure S3A). The introduction of gy‐1 significantly enhanced the binding affinity of Raw264.7^gy‐1‐EGFP^ to PSMA, confirming that gy‐1 expressed on Raw264.7^gy‐1‐EGFP^ membranes has binding activity (Figure 2C). This is a prerequisite for endowing macrophage membrane‐based nanomedicines with improved PCa targetability.

Subsequently, multifunctional core‐shell FeAuNPs were synthesized according to previous reports [20]. The core‐shell structure of FeAuNPs was identified by elemental mapping analysis (Supplementary Figures S4A and B). Then, FeAuNPs were wrapped with cell membranes isolated from Raw264.7^gy‐1‐EGFP^ to yield P‐MMCNPs. Transmission electron microscopy (TEM) and scanning electron microscopy (SEM) images shown in Figure 2D and Supplementary Figure S4C revealed that FeAuNPs were highly uniform spheres and the FeAuNPs core was homogeneously coated with the plasma membrane, which was consistent with the changes in the diameter and zeta potential (Figure 2E). The stability of P‐MMCNPs under simulated physiological conditions was also evaluated. The hydrodynamic diameter of P‐MMCNPs after incubation in serum for 72 h was close to that at 0 h, suggesting that P‐MMCNPs have good applicability for in vivo application and clinical translation (Supplementary Figure S4D). Furthermore, the presence and functional binding activity of macrophage membrane proteins and gy‐1‐EGFP fusion protein in P‐MMCNPs confirmed the successful construction of P‐MMCNPs (Supplementary Figure S3B).

We subsequently explored the feasibility of utilizing P‐MMCNPs as a drug carrier for chemotherapy. DM1 has been adopted as our investigation model due to its significant anti‐proliferative effect, excellent drug loading efficiency, and high compatibility and effectiveness with the targeted drug delivery system [21]. As shown in Supplementary Table S1, P‐MMCNPs exhibited remarkable DM1 encapsulation efficiency and loading capacity. Furthermore, P‐MMCNPs@DM1 displayed glutathione (GSH)‐responsive drug release, aligning perfectly with the drug release requirements in PCa cells characterized by elevated GSH levels (Supplementary Figure S5). This finding underscores the potential of P‐MMCNPs as a promising carrier that can optimize therapeutic efficacy against PCa while minimizing adverse effects. Additionally, we evaluated P‐MMCNPs‐mediated photothermal conversion capability under continuous laser irradiation (Supplementary Figure S6). P‐MMCNPs displayed concentration‐ and power‐dependent photothermal behavior and excellent photothermal stability, making them favorable for tumor PTT applications. Meanwhile, P‐MMCNPs exhibited good MRI and CT capabilities in vitro (Supplementary Figure S7), which provided the opportunity to evaluate the targeting performance of P‐MMCNPs through multimodality imaging. Indeed, the nanocore of P‐MMCNPs is interchangeable and can flexibly encapsulate multiple functional components such as imaging reagents, photodynamic therapy reagents, chemotherapeutic agents, and immunotherapy reagents to meet different needs [22, 23, 24], which emphasizes the great potential of P‐MMCNPs as a versatile platform to achieve precision diagnosis and combination therapy of tumors.

### 2.2 Cellular Targeting and Internalization of P‐MMCNPs

**FIGURE 3:**
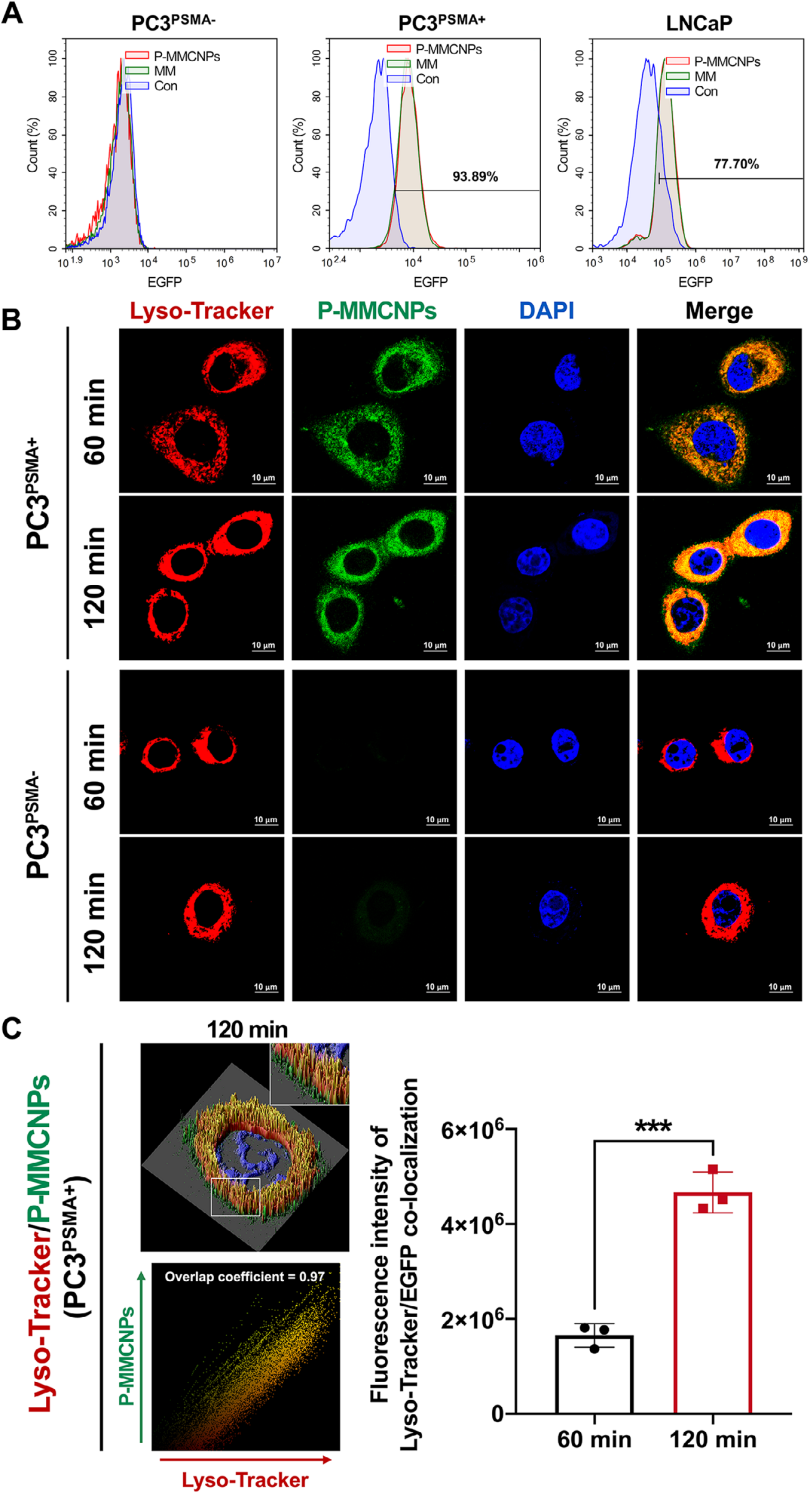
P‐MMCNPs specifically bind with and internalize into PSMA^+^ PCa cells. (A) Flow cytometry to examine the specific binding of P‐MMCNPs to PSMA^+^ PCa cells, (B) Immunofluorescent staining to show the co‐localization of P‐MMCNPs and lysosomes in PSMA^+^ PCa cells. Lysosome was stained with Lyso‐Tracker (red). Scale bar, 10 µm, and (C) Quantitative analysis of the fluorescent intensity ratio of Lyso‐Tracker to EGFP in PSMA^+^ PCa cells. Data were analyzed using the student *t*‐test (two‐tailed). Data were shown as mean ± SD from three independent experiments. ^***^ *P* < 0.001.

Having successfully fabricated P‐MMCNPs, we next investigated whether P‐MMCNPs with TM‐expressed gy‐1 could inherit the prominent PSMA‐targeting ability and tumor cell‐specific internalization of gy‐1. The flow cytometry results show that both P‐MMCNPs and Raw264.7^gy‐1‐EGFP^ macrophage membrane (MM) were able to specifically bind to PSMA‐positive PCa cell lines (PC3^PSMA+^ and LNCaP) rather than PSMA‐negative PC3^PSMA−^ cells (Figure 3A). In contrast to the superior PSMA‐targeting capability of P‐MMCNPs, MMCNPs fabricated with Raw264.7^EGFP^ macrophage membranes (TM‐expressing EGFP but not gy‐1) failed to distinguish PC3^PSMA+^ from PC3^PSMA−^ cells (Supplementary Figure S8), suggesting that TM‐expressed gy‐1 has a critical impact on enhancing tumor targeting and cellular uptake of P‐MMCNPs.

To further confirm the specific targeting of P‐MMCNPs to PC3^PSMA+^ cells, we utilized both PC3^PSMA+^ and PC3^PSMA−^ cells for immunofluorescence confocal assays. As shown in Supplementary Figure S9, P‐MMCNPs rapidly bound to PC3^PSMA+^ cells within 15 min and were internalized by the cells within 30 min. Furthermore, the internalized P‐MMCNPs co‐localized with lysosomes, which increased in a time‐dependent manner (Figures 3B and C). We also employed a human full antibody (PSMAb) having the same binding site as gy‐1 to assess the binding site specificity of P‐MMCNPs. As shown in Supplementary Figure S10, the binding/internalization process of P‐MMCNPs was almost completely inhibited by the pretreatment of PC3^PSMA+^ cells with PSMAb, conclusively demonstrating that the exceptional active targeting capability of P‐MMCNPs is indeed mediated by gy‐1.

Actually, several strategies have been introduced for targeting modification of MMCNPs [25, 26]. Ligand attachment is a simple and efficient way for MMCNPs targeting functionalization, but the ligand anchored to the plasm is supposed to be transient and may detach after a certain period [27, 28]. Also, ligands that are too large or too densely packed on the MMCNPs surface can cause a non‐cooperative effect and non‐specific binding to unwanted cells [29, 30]. Chemical conjugation is another commonly used strategy for MMCNP targeting modification, whereas chemical reactions may alter the surface charge and hydrophobicity, occupy the functional groups of the membrane, or even induce membrane protein denaturation, thus substantially impairing the biocompatibility of MMCNPs [31]. In this study, we employ a genetic engineering strategy to fabricate P‐MMCNPs with TM‐expressing targeting molecules based on the natural protein expression process of macrophages through lentiviral infection, which is expected to be a more rational and stable approach for MMCNP optimization [32, 33]. In addition to PSMA, there are many active molecules expressed on the surface of tumor cells, like epidermal growth factor receptor [34], human epidermal growth factor receptor 2 [35], and glypican‐3 [36]. Therefore, by TM‐expressing the corresponding antibodies or specific ligands on macrophages or other source cells, the P‐MMCNPs platform could be further expanded for targeting other cancers, such as lung cancer, breast cancer, and hepatocellular carcinoma.

### 2.3 In Vitro Evaluation of Immunological Properties of P‐MMCNPs

**FIGURE 4:**
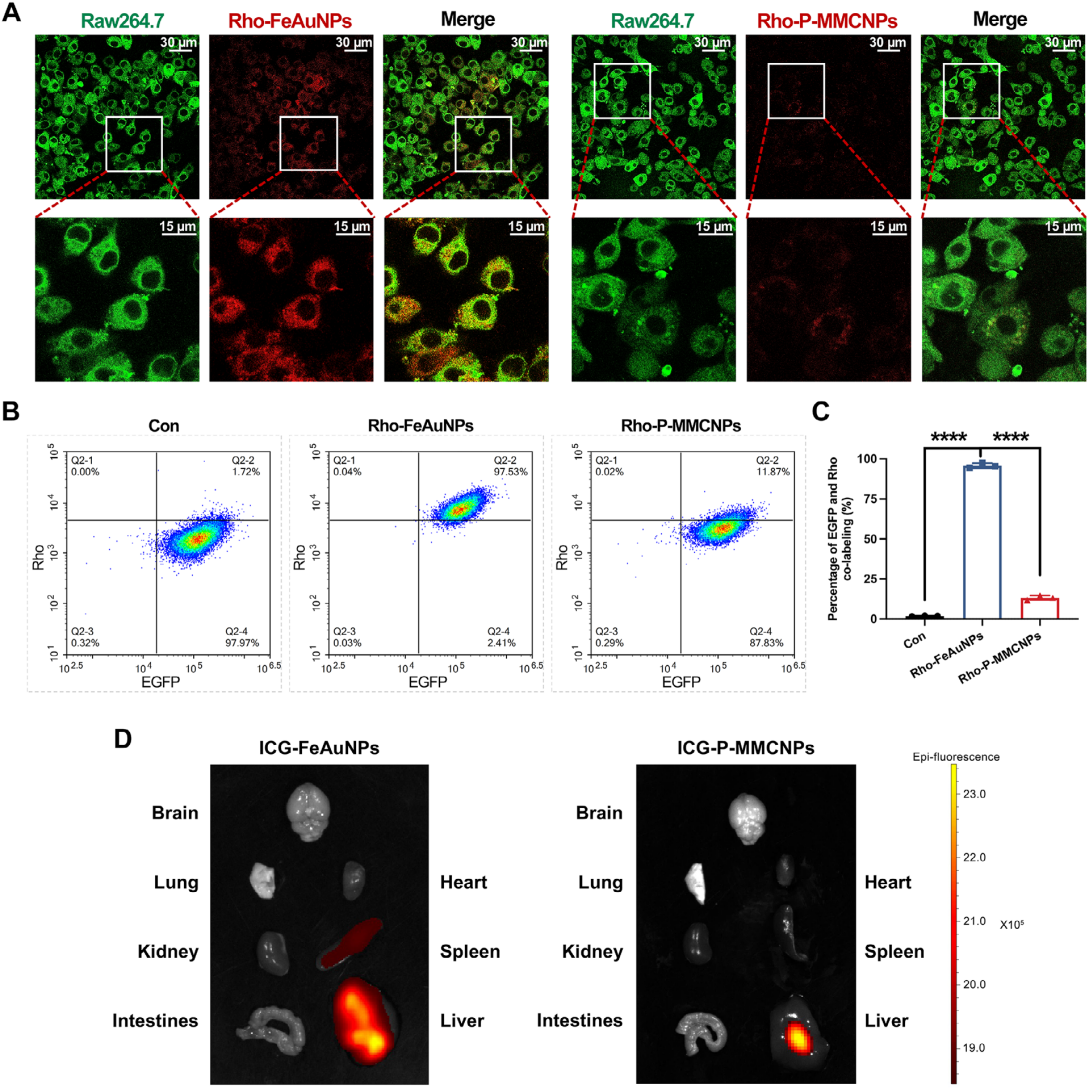
Evaluation of the immune escape ability of P‐MMCNPs. (A) Confocal microscopy to show that P‐MMCNPs escape from phagocytosis by macrophages in vitro. Macrophages were Raw264.7^EGFP^ (green). FeAuNPs and P‐MMCNPs were loaded with Rho‐PEG‐SH (red). Scale bars, 30 µm and 15 µm, (B) Flow cytometry to show that P‐MMCNPs escape from phagocytosis by macrophages in vitro, (C) Quantification of the flow cytometry data in (B), and (D) In vivo distribution of ICG‐FeAuNPs and ICG‐P‐MMCNPs in normal mice at 24 h post‐intravenous injection. Data were analyzed using the student *t*‐test (two‐tailed). Data were shown as mean ± SD from three independent experiments. ^****^ *P* < 0.0001.

Cell membrane‐coated nanoparticles can inherit functional proteins from the source cells, such as CD47 that avoids cellular phagocytosis, and thus can be used to prolong the circulation time of nanomedicines in the bloodstream [37]. To verify whether Raw264.7^gy‐1‐EGFP^ membrane coating could endow P‐MMCNPs with the capacity to avoid immune clearance, rhodamine (Rho)‐FeAuNPs and Rho‐P‐MMCNPs were fabricated. The CLSM images in Figure 4A showed that Rho‐P‐MMCNPs escaped phagocytosis by macrophages more effectively than Rho‐FeAuNPs. Flow cytometry measurement further confirmed that 97.53% of Rho‐FeAuNPs were phagocytized by macrophages versus 11.87% for Rho‐P‐MMCNPs (Figures 4B and C), firmly demonstrating that Raw264.7^gy‐1‐EGFP^ membrane coating did confer P‐MMCNPs enhanced immune evasion capability. Encouraged by the excellent results in vitro, we assessed the in vivo distribution of P‐MMCNPs by loading PEGylated indocyanine green (ICG) for fluorescence tracking. As shown in Figure 4D, P‐MMCNPs demonstrated a remarkable ability to evade immune clearance mechanisms in the liver and spleen of normal mice, a feature that is anticipated to prolong their residence time within the bloodstream and consequently enhance their efficacy in targeting tumor sites.

Furthermore, the immunomodulatory potential of P‐MMCNPs was investigated. To verify whether P‐MMCNPs could re‐educate M2 macrophages by neutralizing tumor‐promoting cytokines secreted by M2 macrophages, ELISA was used first to measure the concentrations of representative cytokines, including TGF‐β, IL‐4, and IL‐10, after pretreatment with P‐MMCNPs for 2 h. As shown in Supplementary Figure S11A, P‐MMCNPs pretreatment significantly decreased the amounts of TGF‐β, IL‐4, and IL‐10, indicative of the cytokine‐neutralization capability of P‐MMCNPs endowed by the Raw264.7^gy‐1‐EGFP^ membrane coating. As a result, cytokine‐induced M2 macrophage polarization was greatly inhibited by P‐MMCNPs (Supplementary Figure S11B). Collectively, our results suggest that P‐MMCNPs have great potential to effectively ameliorate the M2 macrophage‐associated immunosuppressive tumor microenvironment (TME), which is crucial for improving anti‐tumor efficacy.

### 2.4 Active Targeting Ability of P‐MMCNPs to Primary PCa After Systematic Injection

**FIGURE 5:**
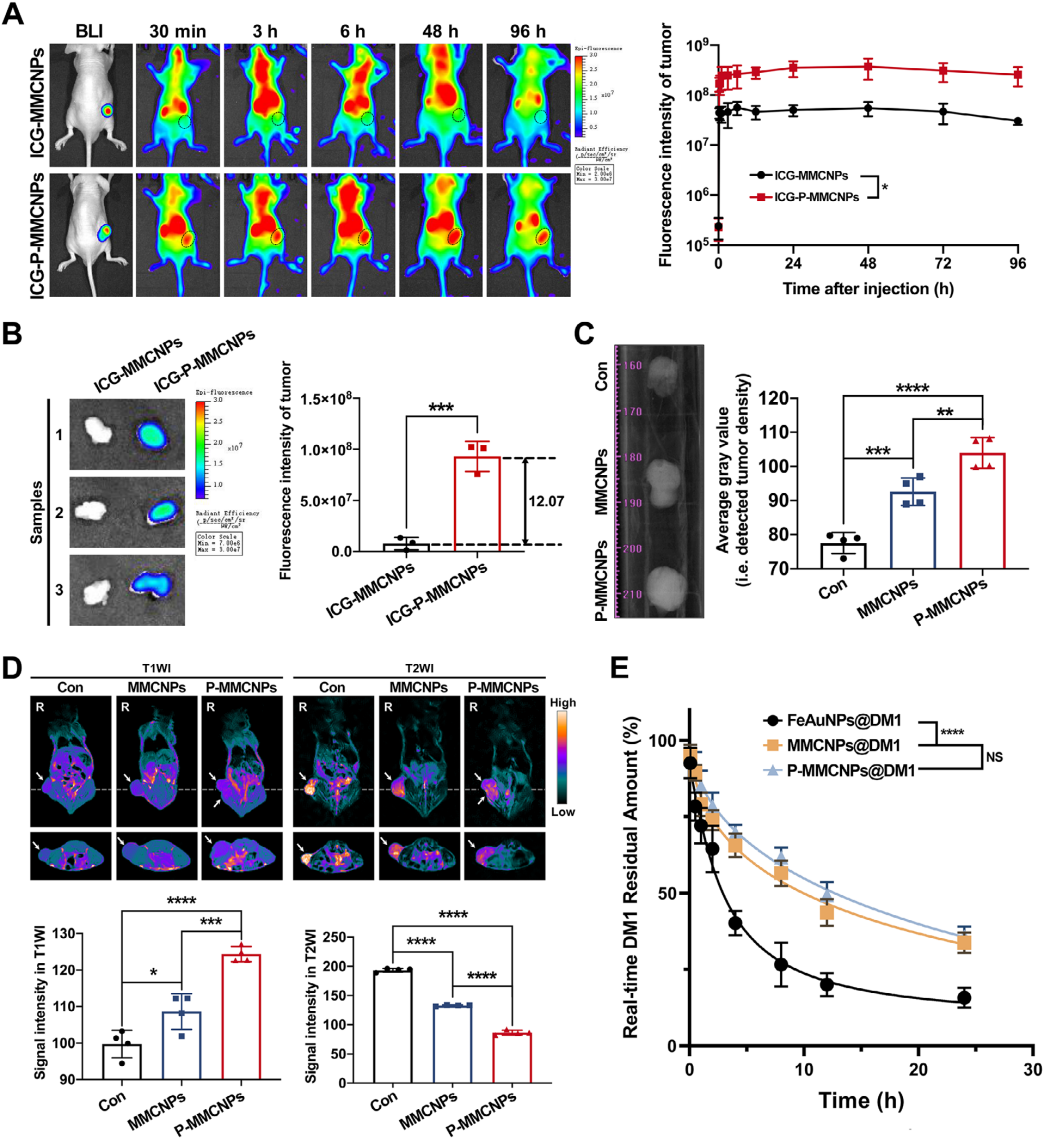
P‐MMCNPs specifically target PSMA^+^ primary PCa in vivo. (A) Fluorescence imaging and quantification of fluorescence intensity to show the specific localization of P‐MMCNPs in subcutaneous PC3^PSMA+^ tumor‐bearing mice. (*n* = 3). Bioluminescence imaging (BLI) displayed the tumor site. Data were analyzed using repeated‐measures ANOVA, (B) Fluorescence imaging and quantification of fluorescence intensity to show the specific localization of P‐MMCNPs in isolated PC3^PSMA+^ tumors. (*n* = 3). Data were analyzed using the student *t*‐test (two‐tailed), (C) CT imaging and quantitative analysis to show the specific localization of P‐MMCNPs in isolated PC3^PSMA+^ tumors. (*n* = 4). Untreated tumor‐bearing mice were used as controls. Data were analyzed using the student *t*‐test (two‐tailed), (D) Dual‐modality MRI and quantitative analysis to show the specific localization of P‐MMCNPs in subcutaneous PC3^PSMA+^ tumor‐bearing mice. (*n* = 4). The white arrow indicates the tumor site. Data were analyzed using the student *t*‐test (two‐tailed), and (E) The half‐life of P‐MMCNPs@DM1 in circulation after intravenous injection. Data were analyzed using repeated‐measures ANOVA. Data were shown as mean ± SD. ^****^ *P* < 0.0001, ^***^ *P* < 0.05, ^**^ *P* < 0.01, and ^*^ *P* < 0.001; **Abbreviation**: NS, no significant.

We further assessed the PCa‐targeting ability of P‐MMCNPs in vivo. As shown in Figure 5A, the luciferase‐expressing PC3^PSMA+^ (PC3^PSMA+^‐luc) subcutaneous tumor was delineated with strong fluorescence even 96 h after intravenous injection of ICG‐P‐MMCNPs, indicating its appreciable tumor specificity and blood circulation. In contrast, the fluorescence signal was barely visible at the tumor focus for ICG‐MMCNPs. The excellent tumor recognition capability of ICG‐P‐MMCNPs could also be confirmed by ex vivo imaging of the tumors collected from mice that were sacrificed 96 h post‐injection (Figure 5B and Supplementary Figure S12). The quantitative analysis revealed a 12‐fold accumulation of ICG‐P‐MMCNPs at the tumor site compared to that of ICG‐MMCNPs, which was consistent with the in vivo results. Furthermore, the absence of ex vivo fluorescence signals in the kidney contradicts the in vivo imaging results (Supplementary Figure S12). Given that we perfused the organs with PBS before isolation to eliminate fluorescent signals from the bloodstream, we speculate that P‐MMCNPs did not truly penetrate the renal tissue but instead remained within the renal vasculature. Similar phenomena were also observed in other blood‐rich organs such as the heart, lungs, liver, and spleen (Supplementary Figure S12).

In addition to fluorescence imaging, P‐MMCNPs‐based CT imaging showed that P‐MMCNPs significantly increased the density of PCa compared to MMCNPs (Figure 5C). Meanwhile, P‐MMCNPs‐based MRI‐T1WI‐positive imaging and MRI‐T2WI‐negative imaging showed that P‐MMCNPs were able to efficiently modulate PCa signaling in vivo with significant specificity and selectivity (Figure 5D). Using EGFP fluorescent tracing, CLSM analysis showed that P‐MMCNPs were specifically internalized into PC3^PSMA+^ cells within subcutaneous tumors, whereas MMCNPs showed barely any cell binding/internalization (Supplementary Figure S13), demonstrating that P‐MMCNPs with superior PSMA‐binding affinity specifically promote their internalization into PCa^PSMA+^ cells in vivo, which is a prerequisite for precision diagnosis and targeted tumor therapy.

The potential impact of a prolonged circulatory half‐life on the targeted accumulation of nanomedicines in vivo has prompted us to conduct pharmacokinetic studies. As depicted in Figure 5E, the circulatory half‐life of P‐MMCNPs@DM1 was significantly extended to 10 h, similar to that of MMCNPs@DM1, but in sharp contrast to the 3.0 h of FeAuNPs@DM1. This underscores that macrophage membrane coating effectively hinders the clearance of both P‐MMCNPs@DM1 and MMCNPs@DM1 from the bloodstream, and that the enhanced targeting ability of P‐MMCNPs relative to MMCNPs is independent of their half‐lives. Collectively, Raw264.7^gy‐1‐EGFP^ membrane ingeniously combines the advantages of macrophage membrane and gy‐1, endowing P‐MMCNPs with enhanced tumor specificity and immune evasion‐mediated blood circulation.

### 2.5 Active Targeting Ability of P‐MMCNPs to PCa Metastasis

Having demonstrated the high targeting capability of P‐MMCNPs to primary PCa, we further explored their feasibility for actively targeting PCa bone metastases, which are the leading cause of death in patients with advanced metastatic PCa [38]. The PCa bone metastasis model was constructed by injecting PC3^PSMA+^‐luc cells into the mouse bone marrow cavity of the tibia via the knee joint (Supplementary Figure S14A). ICG‐P‐MMCNPs and ICG‐MMCNPs were intravenously injected into PC3^PSMA+^‐luc metastatic tumor‐bearing mice. As shown in Supplementary Figure S14B, the fluorescence intensity of the tumor in the ICG‐MMCNPs group decreased rapidly by 1 h post‐injection. In contrast, after ICG‐P‐MMCNPs injection, the fluorescence at the tumor site gradually intensified at 1 h and 12 h, indicating that the efficient enrichment of ICG‐P‐MMCNPs in PCa metastases is attributed to gy‐1‐mediated PSMA recognition. Surprisingly, micro‐metastases with a diameter of 3 mm in the groin next to the bone metastasis were effectively identified by ICG‐P‐MMCNPs instead of ICG‐MMCNPs after excluding the interference from the urine signal (Supplementary Figure S14C). Such striking metastasis targeting was also confirmed by ex vivo imaging of tibial metastases (Supplementary Figure S14D). Compared to the fluorescence signals of ICG‐MMCNPs, ICG‐P‐MMCNPs could lead to a more than 9‐ and 7‐fold increase in signals at micro‐metastatic foci and tibia metastatic, respectively. Therefore, P‐MMCNPs have the diagnostic function of PCa primary foci, metastatic foci, and microscopic foci, which is expected to be used for precise diagnosis and early diagnosis of PCa.

### 2.6 Anti‐Tumor Effects of P‐MMCNPs‐Based Nanomedicine In Vitro

**FIGURE 6:**
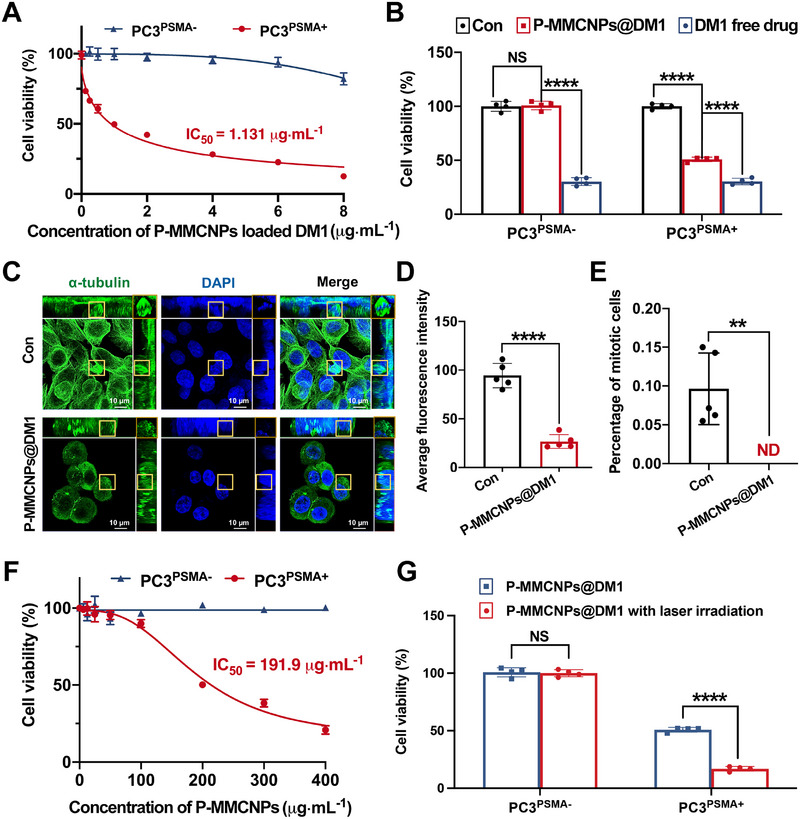
P‐MMCNPs@DM1 alone or combined with PTT show potent cytotoxicity to PSMA^+^ PCa cells in vitro. (A) CCK‐8 assay to show the specific cytotoxicity of P‐MMCNPs@DM1 on PSMA^+^ PCa cells, (B) CCK‐8 assay to show the specific cytotoxicity of P‐MMCNPs@DM1 on PSMA^+^ PCa cells compared with DM1. Untreated PCa cells were used as a control. Data were analyzed using the student *t*‐test (two‐tailed), (C) Immunofluorescent staining of α‐tubulin in PC3^PSMA+^ cells after incubation with P‐MMCNPs@DM1. Untreated PC3^PSMA+^ cells were used as a control. The yellow rectangle indicates mitotic cells or damaged tubulin. Scale bars: 10 µm, (D) Quantification of α‐tubulin fluorescence intensity in (C). Data were analyzed using the student *t*‐test (two‐tailed), (E) Quantification of mitotic cells in (C). Data were analyzed using the student *t*‐test (two‐tailed), (F) CCK‐8 assay to show the photothermal cytotoxicity of P‐MMCNPs on PSMA^+^ PCa cells after laser irradiation, and (G) CCK‐8 assay to show the DM1 and PTT combined anti‐tumor effects of P‐MMCNPs@DM1 on PSMA^+^ PCa cells after laser irradiation. The laser intensity was 0.5 W*·*cm^−2^ and the irradiation time was 5 min. Data were analyzed using the student *t*‐test (two‐tailed). Data were shown as mean ± SD from three independent experiments. ^****^ *P* < 0.0001 and ^**^ *P* < 0.01; **Abbreviations**: ND, not detected; NS, no significant.

DM1 is a potent inhibitor of microtubule assembly with an anti‐mitotic effect at sub‐nanomole concentrations, making it promising for tumor chemotherapy [39]. Although DM1 has been widely explored in cancer treatment, the risk of systemic toxicity remains a significant concern [21]. Thus, the possibility of using P‐MMCNPs as a drug carrier for the targeted delivery of DM1 was investigated. Thiol‐containing DM1 can be incorporated into P‐MMCNPs through Au‐S bonds (P‐MMCNPs@DM1), and the cytotoxicity of P‐MMCNPs@DM1 toward PC3^PSMA+^ and PC3^PSMA−^ cells was evaluated using the CCK‐8 assay. As shown in Supplementary Figure S15, P‐MMCNPs@DM1 significantly killed PC3^PSMA+^ cells, while MMCNPs@DM1 and FeAuNPs@DM1 displayed either moderate or negligible killing effects. Furthermore, P‐MMCNPs@DM1 showed highly targeted cytotoxicity against PC3^PSMA+^ cells with an IC50 of 1.131 µg*·*mL^−1^ for loaded‐DM1, while the cytotoxicity of non‐PSMA‐targeted free DM1 was comparable between the PC3^PSMA+^ and PC3^PSMA−^ two groups (Figures 6A and B). Intriguingly, P‐MMCNPs@DM1 exhibited weaker cytotoxicity toward PC3^PSMA+^ cells compared to free DM1. We posit that free DM1 readily traverses cell membranes and directly kills cells. In contrast, P‐MMCNPs@DM1 necessitates PSMA binding, cellular internalization, and subsequent drug release to exert its cytotoxic effects, suggesting that when the DM1 content is equivalent in both the DM1 free drug and P‐MMCNPs@DM1, the latter may exhibit reduced killing potency. Nonetheless, the specific killing effect of P‐MMCNPs@DM1 offers a safe and effective therapeutic approach for PCa, effectively mitigating the toxic side effects associated with free DM1.

Given that the cytotoxicity of DM1 is closely related to microtubule assembly, we observed immunofluorescence staining on PC3^PSMA+^ cells after P‐MMCNPs@DM1 treatment. As a result, tubulin was severely damaged and mitosis of PC3^PSMA+^ cells was inhibited by P‐MMCNPs@DM1 (Figure 6C–E). Additionally, P‐MMCNPs displayed notable photothermal ablation efficacy on PC3^PSMA+^ cells with an IC50 of 191.9 µg*·*mL^−1^ (Figure 6F). After receiving laser irradiation, the cytotoxicity of P‐MMCNPs@DM1 (200 µg*·*mL^−1^ P‐MMCNPs loaded with 1.2 µg*·*mL^−1^ DM1) was further improved (Figure 6G). Collectively, P‐MMCNPs@DM1 can combine PTT and DM1 chemotherapy to further enhance the killing effect on PCa cells.

### 2.7 Anti‐Tumor Effects of P‐MMCNPs@DM1 In Vivo

**FIGURE 7:**
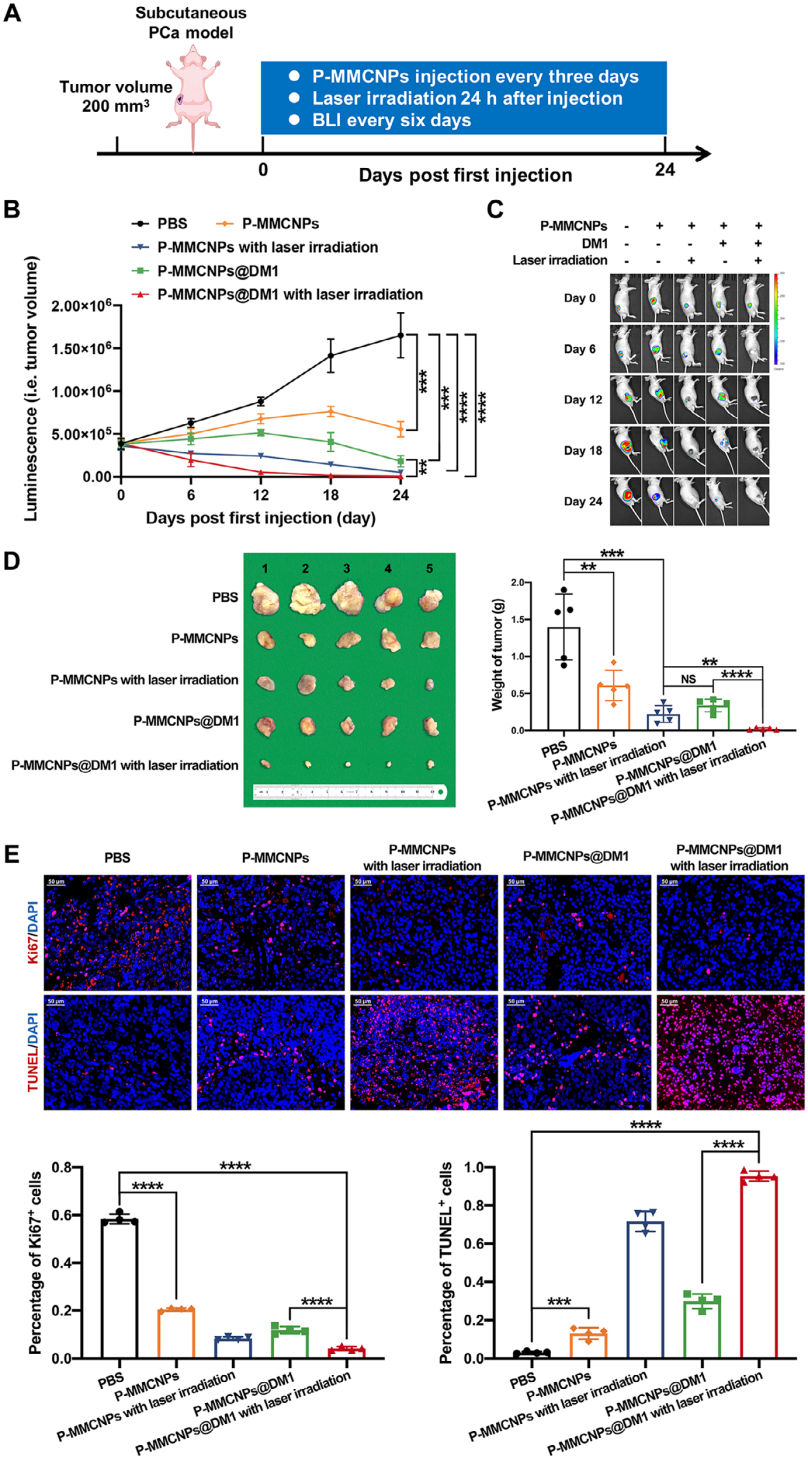
P‐MMCNPs@DM1 in combination with PTT exerts a combined anti‐tumor effect on PSMA^+^ tumors in vivo. (A) Schematic illustration to show the design of animal experiments in the subcutaneous PCa model, (B) Growth curve of PC3^PSMA+^ tumors after indicated treatments. Data were analyzed using repeated‐measures ANOVA, (C) Representative BLI to show the tumor growth after indicated treatment, (D) Picture of isolated tumors and tumor weights after mice were sacrificed at endpoints. (*n* = 5). Data were analyzed using the student *t*‐test (two‐tailed), and (E) Immunofluorescent staining and quantitative analysis of Ki‐67 and TUNEL staining in the tumor tissues after the indicated treatment. Data were analyzed using the student *t*‐test (two‐tailed). Scale bar, 50 µm. Data were shown as mean ± SD. ^****^ *P* < 0.0001, ^***^ *P* < 0.01, and ^**^ *P* < 0.001; **Abbreviation**: NS, no significant.

A schematic illustration showed the design of animal experiments to evaluate the combined therapeutic efficacy of P‐MMCNPs@DM1 (Figure 7A). PC3^PSMA+^‐luc‐bearing BALB/c nude mice were randomly divided into five groups: (1) intravenous injection with PBS as a control, (2) intravenous injection with P‐MMCNPs without laser irradiation, (3) intravenous injection with P‐MMCNPs followed by 808 nm laser irradiation, (4) intravenous injection with P‐MMCNPs@DM1 without irradiation, and (5) intravenous injection with P‐MMCNPs@DM1 followed by 808 nm laser irradiation. Tumor growth was monitored using a Xenogen IVIS in vivo imaging system for bioluminescence detection. In contrast to the continuous tumor growth in the PBS group, all four other groups showed significant inhibition of tumor growth (Figures 7B–D and Supplementary Figure S16). It is noteworthy that the injection of P‐MMCNPs alone significantly suppressed tumor progression compared with the PBS group, suggesting that macrophage membrane‐mediated cytokine neutralization and macrophage polarization reprogramming may exert anti‐tumor efficacy in vivo. Compared to P‐MMCNPs@DM1 without irradiation, the integration of DM1 with PTT exerted stronger anti‐tumor efficacy during the entire treatment period, strongly demonstrating the competence of the P‐MMCNPs‐based platform for PCa‐specific and combined therapy. Meanwhile, we conducted additional studies to comparatively evaluate the therapeutic efficacy of FeAuNPs@DM1, MMCNPs@DM1, and P‐MMCNPs@DM1 in the PC3^PSMA+^ subcutaneous xenograft model. As depicted in Supplementary Figure S17, P‐MMCNPs@DM1 significantly inhibited tumor growth, while MMCNPs@DM1 and FeAuNPs@DM1 displayed either moderate or negligible anti‐tumor effects.

The excellent anti‐PCa efficacy of P‐MMCNPs‐based nanomedicine was further supported by Ki67 immunohistochemistry and TUNEL staining (Figure 7E). It can be seen clearly that all treatment modalities, even with P‐MMCNPs only, strongly suppressed tumor cell proliferation and efficiently induced cell apoptosis. The combined treatment using DM1 and PTT showed dramatically improved anti‐tumor efficacy.

**FIGURE 8:**
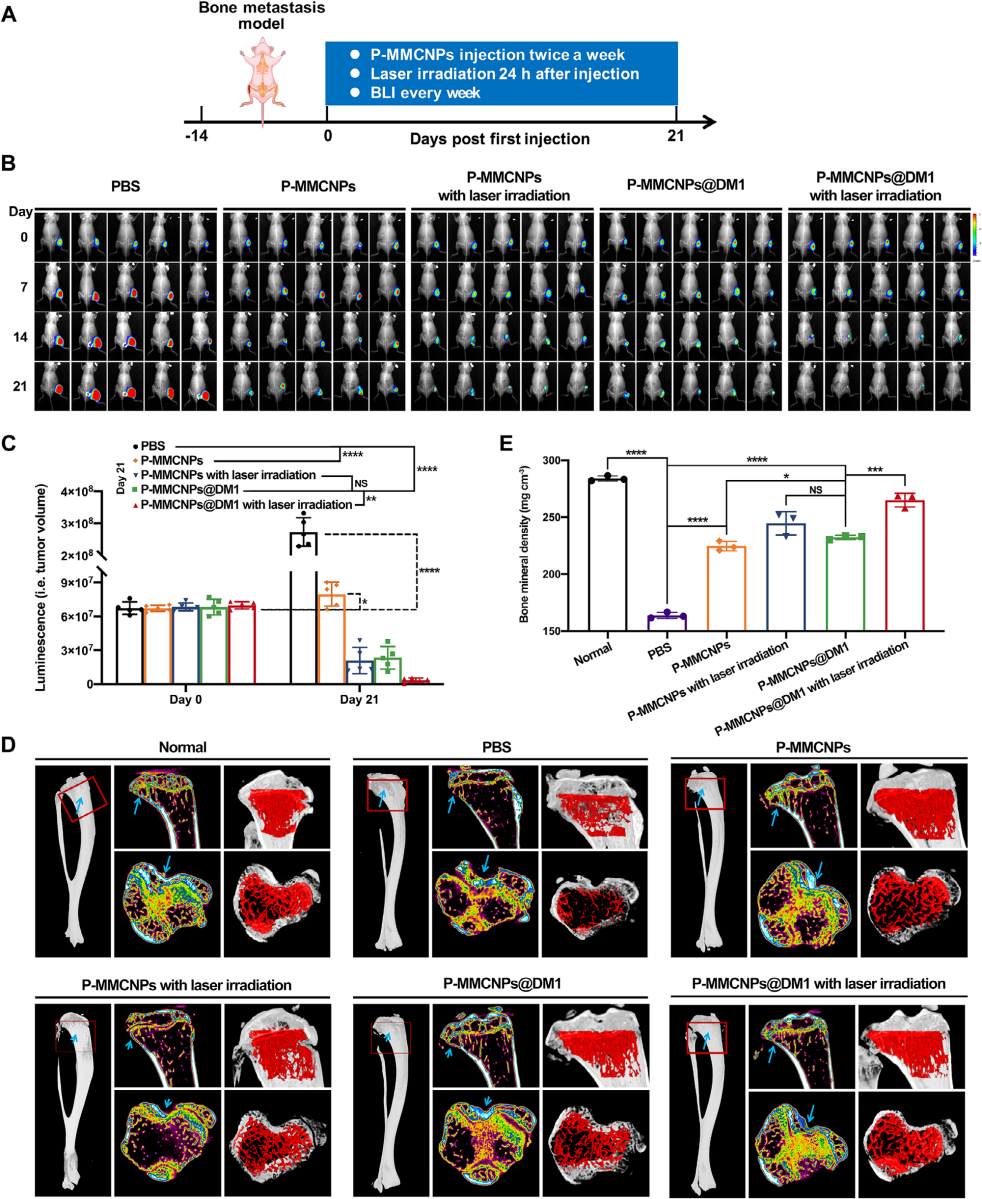
P‐MMCNPs@DM1 in combination with PTT exerts a combined anti‐tumor effect in the PSMA^+^ PCa bone metastasis model. (A) Schematic illustration to show the design of animal experiments in the PCa bone metastasis model, (B) BLI to show the growth of bone metastatic tumor after indicated treatment, (C) Quantification of the fluorescence intensity in (B). Data were analyzed using the student *t*‐test (two‐tailed), (D) Micro‐CT imaging of the tibia with a metastatic tumor. Tumor‐free BALB/c nude mice were used as normal, and (E) Bone mineral density of the tibia in different groups according to the result in (D). Data were analyzed using the student *t*‐test (two‐tailed). Data were shown as mean ± SD. ^****^ *P* < 0.0001, ^***^ *P* < 0.05, ^**^ *P* < 0.01, and ^*^ *P* < 0.001; **Abbreviation**: NS, no significant.

Active targeting‐mediated anti‐tumor effects of P‐MMCNPs@DM1 were also observed in the PCa bone metastasis model (Figure 8A). Consistently, a combination of DM1 chemotherapy and PTT resulted in optimal therapeutic outcomes, and the metastatic tumors were eliminated after 21‐day treatment (Figures 8B and C). Furthermore, the destruction of bone trabecular microstructure was significantly improved after effective treatment of metastases, as illustrated by the micro‐CT results in Figure 8D. Meanwhile, tumor‐induced loss of bone mineral content was recovered (Figure 8E). To investigate the alterations in osteoblasts and osteoclasts within the metastatic tumor‐bearing tibia following P‐MMCNPs@DM1 treatment, we extended our analysis by fluorescent staining. As shown in Supplementary Figure S18, a notable increase in osteoblasts and a significant decrease in osteoclasts were observed in the combined DM1 and PTT treatment group compared to the control, suggesting that P‐MMCNPs@DM1 is effective in inhibiting tumor‐induced bone destruction. Notably, injection of P‐MMCNPs alone also showed significant anti‐tumor efficacy. Therefore, P‐MMCNPs benefit from their impressive PSMA targetability and exhibit great potential for the successful management of primary prostate tumors, micro‐metastases, and concomitant damages.

### 2.8 Anti‐Tumor Immunomodulation of P‐MMCNPs In Vivo

**FIGURE 9:**
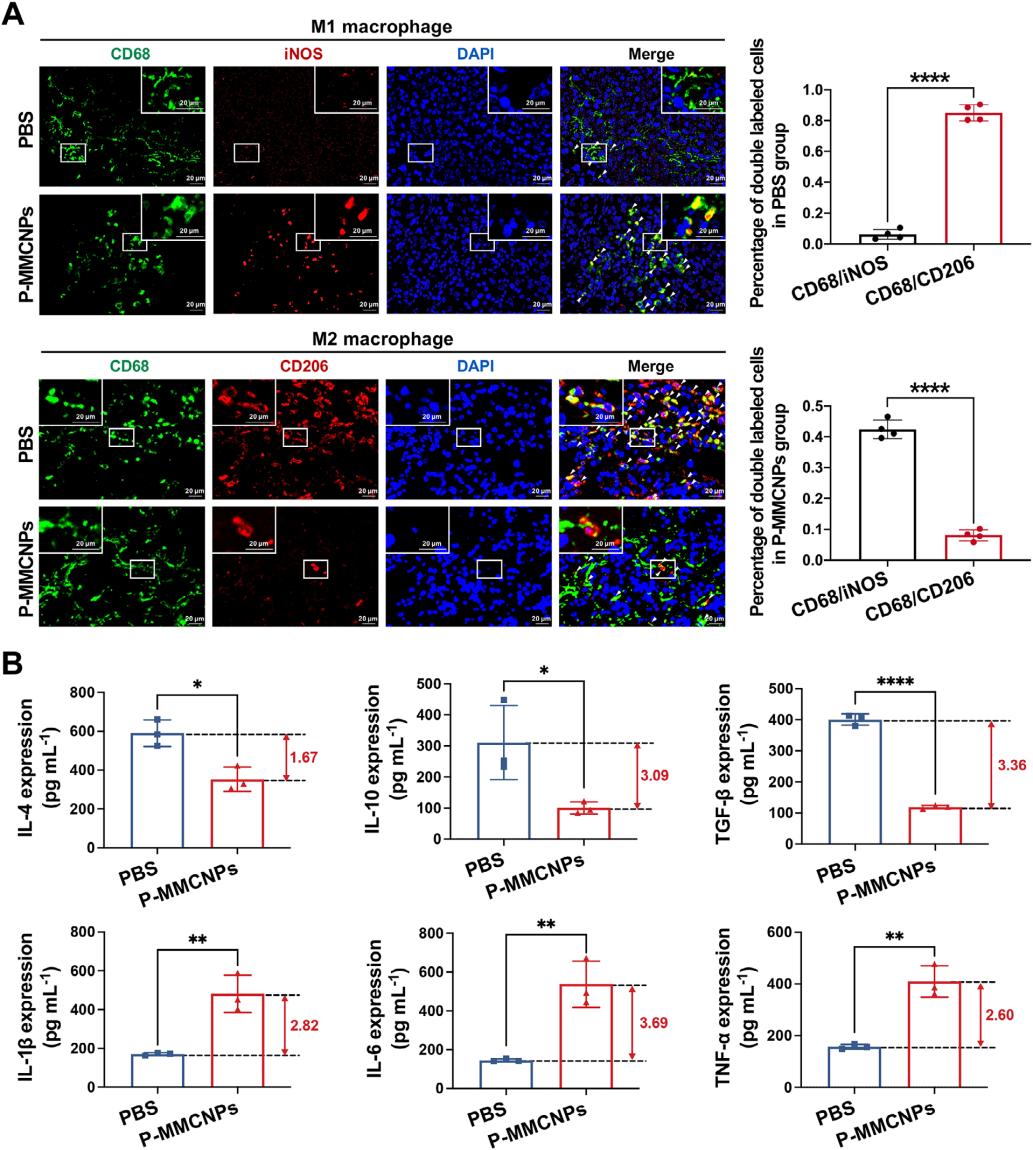
P‐MMCNPs inhibit the growth of PSMA^+^ tumors through macrophage‐associated immunomodulation in vivo. (A) Immunofluorescent staining and quantitative analysis of M1 and M2 macrophages in tumor tissues after P‐MMCNPs treatment. Data were analyzed using the student *t*‐test (two‐tailed). Scale bar, 20 µm and (B) ELISA to examine the levels of different cytokines in tumor tissues. Data were analyzed using the student *t*‐test (two‐tailed). Data were shown as mean ± SD. ^****^ *P* < 0.0001, ^**^ *P* < 0.05, and ^*^ *P* < 0.01.

To gain deeper insight into the inhibitory mechanism of P‐MMCNPs in vivo, we further investigated whether it could exert anti‐tumor effects by blocking M2 macrophage‐related tumor‐promoting cascades. To verify our hypothesis, immunofluorescence staining of macrophages in tumor tissues was first performed. As shown in Figure 9A, a notable reversal in the M1/M2 macrophage ratio was observed in tumors treated with P‐MMCNPs, compared to the PBS‐treated group. Furthermore, there was no significant difference in the number of CD68‐positive cells between the PBS group and the P‐MMCNPs‐treated group (Supplementary Figure S19), suggesting that the alteration in the M1/M2 macrophage ratio mediated by P‐MMCNPs was not attributed to specific toxicity toward M2 macrophages but rather to a modulation of macrophage polarization. We also performed flow cytometry analysis to deeply investigate the ratio of M1 and M2 macrophages in the tumor tissue suspension. As shown in Supplementary Figure S20, a significant increase in CD80/CD86‐marked M1 macrophages and a significant decrease in CD163/CD206‐marked M2 macrophages were detected following P‐MMCNPs treatment, compared to the PBS control group. Then, ELISA was performed to detect representative cytokines secreted by M1 and M2 macrophages in tumor tissues collected from mice after treatment with P‐MMCNPs. As shown in Figure 9B, PBS‐treated tumors exhibited greater amounts of pro‐tumoral cytokines IL‐4, IL‐10, and TGF‐β, which were significantly suppressed in the P‐MMCNPs‐treated group. Meanwhile, those of M1‐related anti‐tumor cytokines IL‐1β, IL‐6, and TNF‐α were elevated in P‐MMCNPs‐treated tumors, which agrees with the histological results described above. These results suggest that P‐MMCNPs can reprogram macrophages from the M2 phenotype to the M1 phenotype and neutralize pro‐tumoral cytokines, thereby effectively regulating TME into an anti‐tumor state. Based on the interaction between cell membrane receptors and different biomolecules, cell membrane‐coated nanomedicines have been widely used for antibody or cytokine neutralization and even to counteract biotoxins. As demonstrated, erythrocyte‐ or platelet‐membrane‐coated nanomedicines can serve as biomimetic baits to neutralize autoantibodies and treat autoimmune hemolytic anemia and immune thrombocytopenia [40, 41, 42, 43, 44]. Macrophage‐derived nanosponges can adsorb pro‐tumoral cytokines for renal cell carcinoma immunotherapy [45]. Notably, M2 macrophage infiltration contributes to the pro‐tumoral environment in many types of cancers [46], indicating the universality of P‐MMCNPs for tumor immunotherapy.

M2 macrophages also stimulate endothelial cell proliferation‐based tumor angiogenesis and epithelial‐mesenchymal transition (EMT)‐mediated tumor metastasis by secreting VEGFC and TGF‐β [46, 47, 48]. Therefore, we further investigated the potential of P‐MMCNPs to inhibit tumor angiogenesis and the EMT process. As shown in Supplementary Figure S21, *VEGFC* expression was downregulated, tumor angiogenesis markers VEGFR2 and CD105 were significantly reduced, and EMT‐associated proteins vimentin and ECAD were significantly reversed after treatment with P‐MMCNPs compared to the PBS group, indicating the strong potential of P‐MMCNPs to effectively prevent tumor progression. It is worth noting that this study used nude mice; although it did not affect the assessment of macrophage‐mediated nanoparticle uptake and macrophage‐related immune regulation, we need to acknowledge the limitations of studying broader adaptive immune responses in this model. The use of syngeneic or humanized mouse models in the follow‐up studies will more accurately reflect clinical immune responses.

### 2.9 Toxicity Evaluation of P‐MMCNPs‐Based Nanoformulations

**FIGURE 10:**
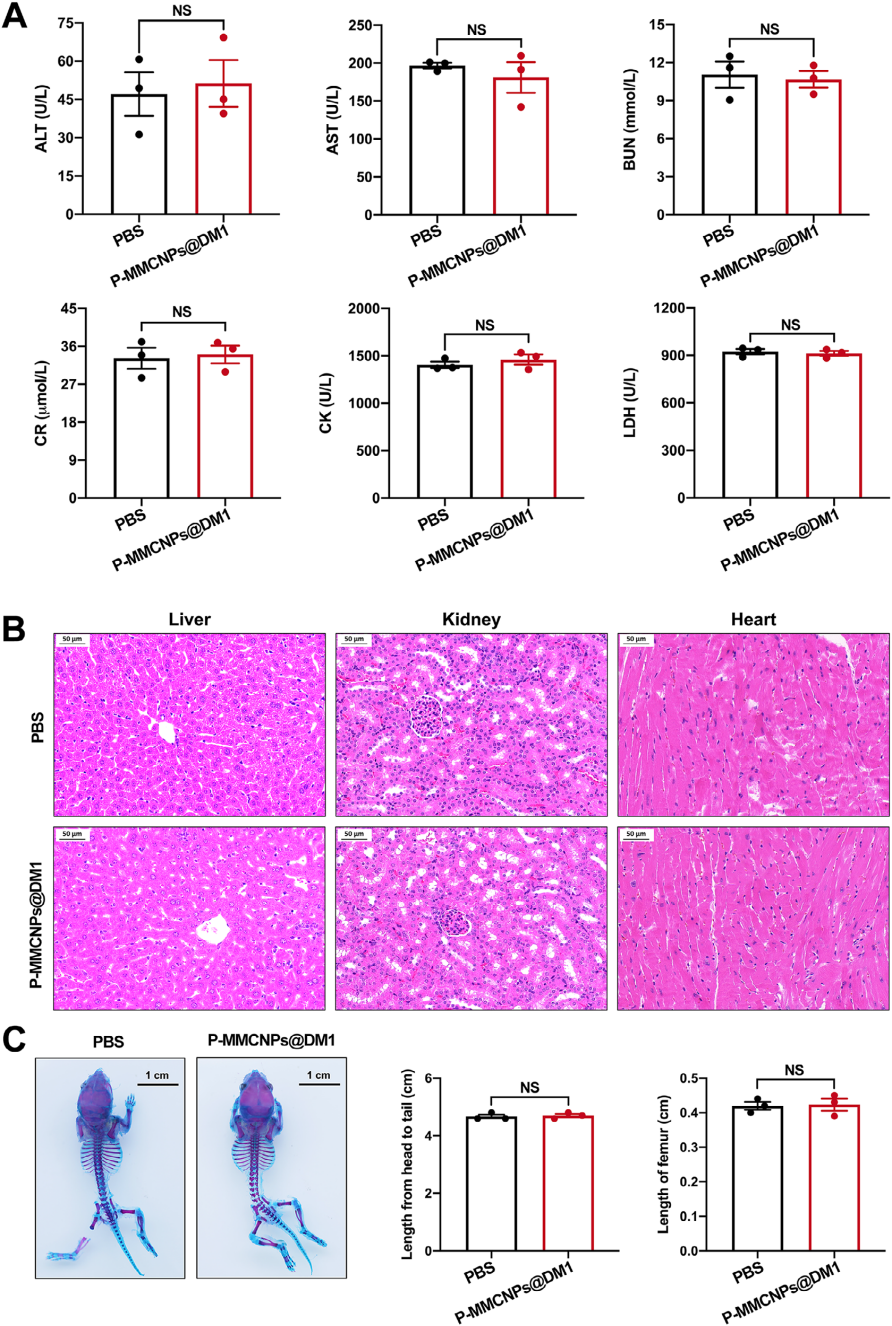
Biosafety evaluation of P‐MMCNPs@DM1 in vivo. (A) Serological test to examine the liver, kidney, and myocardial functions after two consecutive weeks of P‐MMCNPs@DM1 application. Alanine aminotransferase (ALT), aspartate transaminase (AST), and alkaline phosphatase (ALP) are commonly used indexes for evaluating liver function. Blood urea nitrogen (BUN) and serum creatinine (CR) are commonly used indexes for evaluating kidney function. Creatine kinase (CK) and lactate dehydrogenase (LDH) are commonly used index evaluating myocardial function. Data were analyzed using the student *t*‐test (two‐tailed), (B) H&E staining to examine the organ toxicity of P‐MMCNPs@DM1. Scale bar, 50 µm, and (C) Alizarin red/alixin blue staining to examine osteodevelopment after the application of P‐MMCNPs@DM1. Data were analyzed using the student *t*‐test (two‐tailed). Scale bar, 1 cm. Data were shown as mean ± SD. *n* = 3 for each group. **Abbreviation**: NS, no significant.

We evaluated the primary toxicity of P‐MMCNPs‐based nanoformulations in vivo. Normal or pregnant C57 mice were intravenously injected with P‐MMCNPs or P‐MMCNPs@DM1 for 2 weeks and then sacrificed. No obvious physiological abnormalities or systemic toxicity were detected in any of the treated mice in serologic tests (Figure 10A and Supplementary Figure S22A). In addition, the peripheral organ toxicity, neurotoxicity, and embryotoxicity of P‐MMCNPs and P‐MMCNPs@DM1 were comprehensively evaluated. The results showed that no histomorphological abnormalities were observed (Figure 10B and C and Supplementary Figures S22B–F), demonstrating that P‐MMCNPs can be safely applied as an excellent platform for tumor therapy.

## 3 Conclusions

In this study, we present a promising versatile platform, P‐MMCNPs, for PSMA‐targeted multimodal imaging and combined therapy. Several novel aspects were observed in this study. First, instead of using conventional post‐functionalization approaches—such as ligand attachment or chemical conjugation—we employ a genetic engineering strategy. This strategy enables direct expression of a tumor‐specific ligand across the macrophage membrane. As a result, the membrane‐coated P‐MMCNPs gain two key properties: immune escape capability and tumor‐targeting efficiency. Meanwhile, the FeAuNPs used in the construction of P‐MMCNPs serve as a replaceable nanocore, making P‐MMCNPs a promising multifunctional platform. Second, the cargo‐loading capacity of P‐MMCNPs is highly flexible. It allows encapsulation of multiple functional components, such as imaging reagents and chemotherapeutic agents, for diverse clinical applications. Such features underscore the immense potential of P‐MMCNPs as a versatile platform for tumor‐targeted imaging and therapeutics. Here, we have prepared P‐MMCNPs@DM1 by loading the cytotoxic drug DM1 onto P‐MMCNPs. Third, P‐MMCNPs@DM1 demonstrates strong PSMA‐targeted multimodal imaging capabilities. It also exhibits a combined anti‐tumor effect, integrating DM1 chemotherapy and PTT. This dual‐action approach enables successful management of both primary and metastatic prostate tumors. Importantly, it minimizes concomitant damages without inducing obvious systemic toxicity. Fourth, P‐MMCNPs alone can reprogram macrophages from the M2 phenotype to the M1 phenotype and neutralize pro‐tumoral cytokines, thereby effectively regulating TME into an anti‐tumor state.

In summary, our work demonstrates that the innovative P‐MMCNPs are a versatile platform for improving PCa‐targeted diagnostic and therapeutic efficacy and avoiding side effects. Moreover, it holds promise for expanding into the diagnosis and treatment of other diseases, with potential for clinical translation. Additionally, it offers novel insights into integrating nanomedicine‐based targeted therapy with immunomodulation.

## 4 Experimental Section

### 4.1 Methods

#### 4.1.1 Cell Culture and Lentivirus Infection

Murine macrophage Raw264.7 and human PCa cell lines (i.e., PC3^PSMA−^ and LNCaP) were purchased from the American Type Culture Collection. PC3^PSMA+^ cells were constructed in our laboratory based on the PC3^PSMA−^ cell line [17, 18, 19]. The cells were maintained in high‐glucose Dulbecco's Modified Eagle Medium (DMEM), DMEM/F12, and RPMI‐1640 medium supplemented with 10% fetal bovine serum (FBS, Gibco, 10099‐141, USA) and 1% penicillin/streptomycin (Invitrogen, 15140‐122, USA). Cells were cultured at 37°C with 5% CO_2_ in a humidified incubator.

Lentivirus used for Raw264.7^gy‐1‐EGFP^ construction was synthesized based on the GV218 vector (GeneChem, China). Briefly, the extracellular segment of gy‐1 serves as the PSMA targeting portion, while the cytoplasmic domain of EGFP acts as a reporter molecule, anchored to the macrophage membrane with the help of the CD8 hinge domain and CD8 TM region. Three days after lentivirus infection (MOI = 30), Raw264.7^gy‐1‐EGFP^ with gy‐1‐EGFP TM expression and Raw264.7^EGFP^ with EGFP TM expression were screened with 3 µg*·*mL^−1^ puromycin. Then, cells were harvested, washed, and resuspended in fluorescence‐activated cell sorting (FACS) buffer at a density of 1 × 10^6^ cells*·*mL^−1^ for flow cytometry analysis (Beckman Coulter, USA).

#### 4.1.2 Cellular ELISA

To evaluate the binding ability of Raw264.7^gy‐1‐EGFP^ to PSMA, 2 × 10^3^ Raw264.7^gy‐1‐EGFP^ were seeded in a 96‐well plate and incubated overnight for cell attachment. Then, the recombinant PSMA protein with His tag (Sino Biological, 15877‐H07H, China) was added to the plate for incubation. Due to the transmembrane expression of PSMA scFv (gy1) on the extracellular fragment of Raw264.7^gy‐1‐EGFP^, Raw264.7^gy‐1‐EGFP^ can capture recombinant PSMA protein. Subsequently, cells were washed with phosphate‐buffered saline (PBS), incubated with HRP‐conjugated anti‐6×His antibody (Abcam, ab1187, UK), and visualized using 3,3′,5,5′‐tetramethylbenzidine (TMB, Beyotime, P0209, China). The absorbance was measured using a microplate reader at 450 nm (Infinite 200, Tecan, Switzerland). The signal intensity will reflect the binding ability of Raw264.7^gy‐1‐EGFP^ to PSMA. To avoid the result shift caused by the internalization of PSMA protein after binding to gy‐1, we set the incubation parameters of PSMA protein and Raw264.7^gy‐1‐EGFP^ to 1 h at 4°C, as this parameter can inhibit the internalization reaction after protein‐receptor binding [17].

#### 4.1.3 Membrane Extraction

The plasma membrane of Raw264.7^gy‐1‐EGFP^ was collected according to the previously published protocol [49]. Briefly, 1 × 10^7^ Raw264.7^gy‐1‐EGFP^ was washed with PBS three times and resuspended in 1 mL hypotonic lysing buffer containing 20 mM Tris‐HCl (pH 7.5), 10 mM KCl, 2 mM MgCl_2_, and an EDTA‐free protease inhibitor cocktail (Roche, 04693159001). After incubating at 4°C for 15 min, cells were disrupted by gentle sonication on ice (30 s sonication with 30 s pause for 5 min). The homogenized solution was centrifuged at 3500 g for 5 min at 4°C to remove organelles, and the resulting supernatant was centrifuged again at 20,000 g for 30 min at 4°C to collect cell membrane precipitates. The precipitates were washed with DNase‐free/RNase‐free water and stored at −80°C for subsequent studies.

#### 4.1.4 Preparation of P‐MMCNPs

Core‐shell FeAuNPs were prepared by reducing gold chloride trihydrate (HAuCl_4_·3H_2_O, 16961‐25‐4, Aladdin, China) in the presence of pre‐synthesized magnetite nanoparticles [20]. The resulting FeAuNPs were collected magnetically and washed three times with deionized water to remove excess reagents. Then, 200 µg FeAuNPs and 400 µg Raw264.7^gy‐1‐EGFP^ membranes were dissolved in 1 mL DNase‐free/RNase‐free water. The mixture was subjected to ultrasonic treatment in an ice bath with an FS30D ultrasonic instrument (Fisher Scientific, USA) at a frequency of 42 kHz and a power of 100 W for 5 min. The resulting suspensions were extruded serially through 400 nm and 200 nm membrane filters (Whatman, UK) using a mechanical extruder 10 times as previously reported [50].

#### 4.1.5 Characterization of P‐MMCNPs

The structure of P‐MMCNPs was examined by TEM (TECNAI Spirit, FEI). Briefly, a drop of P‐MMCNPs solution at a concentration of 200 µg*·*mL^−1^ was added onto a carbon‐coated copper grid (Electron Microscopy Sciences). Following 10 min of sample deposition, the grid was rinsed with 10 drops of distilled water. A drop of 1% phosphotungstic acid was deposited on the sample, followed by a rinse process with distilled water. The grid was dried naturally and visualized using a 120 kV FEI TEM. The morphology of P‐MMCNPs was determined by SEM. The lyophilized P‐MMCNPs were mounted on a carbon tape and sputter‐coated with gold using a sputter with a current set at 40 mA (Dynavac Mini Coater, Dynavac). The SEM images were obtained using a Philips XL30 SEM with an accelerating voltage of 3 kV. The hydrodynamic size and zeta potential of P‐MMCNPs were measured by dynamic light scattering (DLS) using a Nano Zetasizer (Malvern, UK).

#### 4.1.6 Drug Loading and Release Properties Evaluation in Vitro

FeAuNPs and DM1 (Ruixi Biotech, China) were dispersed in deionized water at various mass ratios, and the mixture was stirred in the dark for a specified period. Following this, the mixture was centrifuged, and the precipitate was resuspended in a defined amount of PBS containing macrophage membrane. The resuspended mixture was then repeatedly extruded through a liposome extruder. After centrifugation, P‐MMCNPs@DM1 were obtained. The supernatants were collected for calculating the encapsulation efficiency and drug loading capacity.

For the in vitro DM1 release experiment, 2 mg of different DM1‐loaded samples were suspended in 2 mL of PBS. These suspensions were then dialyzed against 50 mL PBS (pH 7.4) using a dialysis membrane (MW cut‐off = 5000). The sample was shaken at 120 rpm at 37°C in the dark. At predetermined time points, 2 mL of solution was taken away and replenished with 2 mL of fresh PBS. The amount of DM1 released was detected by high‐performance liquid chromatography (HPLC), and the cumulative release of DM1 was calculated. To investigate the GSH‐sensitive drug release behavior, the drug release of DM1 from P‐MMCNPs@DM1 was completed at 37°C in PBS containing different concentrations of GSH; other steps were consistent with the methods described above.

#### 4.1.7 Photothermal Conversion Evaluation in Vitro

1 mL PBS solutions of P‐MMCNPs with certain concentrations were put into microtubes and irradiated with an 808 nm laser (MDL‐III‐808, Changfu Technology, China) for 10 min. The power density of the laser ranged from 1.0 W*·*cm^−2^ to 3.0 W*·*cm^−2^. The temperature distribution was recorded by using a thermal imaging camera (FLIR, T420, USA) every 30 s. Four cycles of laser on/off were employed to study the photothermal stability of P‐MMCNPs.

#### 4.1.8 Specific Binding Assay

PC3^PSMA−^, PC3^PSMA+^, and LNCaP (PSMA^+^) were harvested, washed, and resuspended in FACS buffer at a density of 1×10^6^ cells*·*mL^−1^. Then, cells were incubated with 200 µg*·*mL^−1^ MM or P‐MMCNPs for 30 min at 4°C under darkness. After washing with FACS buffer, cells were analyzed by flow cytometry (Beckman Coulter, USA). PSMA identification differences between MMCNPs and P‐MMCNPs were also evaluated as described above.

#### 4.1.9 Confocal Microscope Imaging Evaluating Cell Targeting and Internalization

1 × 10^5^ cells (i.e., PC3^PSMA−^ and PC3^PSMA+^ cells) were seeded into confocal dishes. After overnight incubation, the cytomembrane and lysosomes were stained with the tracer according to the manufacturer's instructions (Beyotime Biotechnology, C1036, China; Solarbio, L8010, China). Subsequently, the medium was replaced with fresh medium containing 200 µg*·*mL^−1^ P‐MMCNPs for a certain incubation time. Then, cells were washed with PBS to remove any unbound P‐MMCNPs and fixed with 4% paraformaldehyde. Fluorescence images were obtained using a laser confocal microscope (Olympus, FV31s‐sw, Japan) and analyzed with Image‐Pro Plus.

For targeting and internalization blocking experiments, human full antibody (PSMAb) constructed in our laboratory from gy1 was used [17, 18, 19]. Briefly, 100 nmol*·*mL^−1^ PSMAb were pre‐incubated with PC3^PSMA+^ cells after cytomembrane and lysosome staining. Then, cells were incubated with 200 µg*·*mL^−1^ P‐MMCNPs for 60 min at 37°C under darkness, washed with PBS, and fixed with 4% paraformaldehyde for microscope visualization as described.

#### 4.1.10 Immune Evasion Assay in Vitro

PEGylated rhodamine (Rho‐PEG‐SH, Ruixi Biotech, China) and FeAuNPs were mixed in a stoichiometric ratio of 2:1. The reaction mixture was stirred for 2 h at room temperature for Au‐S bond‐mediated Rho loading. Rho‐P‐MMCNPs and Rho‐FeAuNPs were incubated with Raw264.7^EGFP^ cells for 24 h in the incubator. After washing with PBS, cells were fixed with 4% paraformaldehyde and visualized using a laser confocal microscope (Olympus, FV31s‐sw, Japan). Image‐Pro Plus software was employed for quantification.

To further confirm that P‐MMCNPs could avoid macrophage phagocytosis, Raw264.7^EGFP^ cells were harvested, washed, and resuspended in FACS buffer at a density of 1 × 10^6^ cells*·*mL^−1^. Then, cells were incubated with 200 µg*·*mL^−1^ Rho‐FeAuNPs or Rho‐P‐MMCNPs for 30 min at 4°C under darkness. After washing with FACS buffer, cells were analyzed by flow cytometry (Beckman Coulter, USA).

#### 4.1.11 Cytokine Neutralization and Macrophage Polarization

200 µg*·*mL^−1^ P‐MMCNPs were pre‐incubated with M2 macrophage‐related cytokines (i.e., TGF‐β, IL‐4, and IL‐10, Sino Biological, China) at 37°C for 2 h. Subsequently, cytokine solutions were centrifuged with a speed of 14000 g for 20 min at 4°C to separate P‐MMCNPs and neutralized cytokines. The supernatant was collected, and residual cytokines were quantified with ELISA kits (Proteintech, China). To investigate cytokine neutralization‐mediated polarization regulation, P‐MMCNPs pre‐treated IL‐4 solution (20 ng*·*mL^−1^) was used for Raw264.7 cell culture. Three days later, cells were stained with APC‐conjugated CD206 polyclonal antibody (PA5‐46879, Invitrogen, USA) for flow cytometry detection.

#### 4.1.12 Animal Model Construction

Male BALB/c nude mice and C57 mice were housed in a temperature‐controlled room with free access to food and water. To construct subcutaneous tumor‐bearing mice, 5 × 10^6^ PC3^PSMA+^‐luc cells were subcutaneously injected into the right flank. When tumor volume reached 200 mm^3^, animals were used for follow‐up experiments. For the bone metastasis model, 1 × 10^6^ PC3^PSMA+^‐luc cells were injected into the medullary cavity of the right tibia via the knee joint. Two weeks later, animals were subjected to further studies.

#### 4.1.13 In Vivo Fluorescence Imaging

ICG‐PEG‐SH (MW = 2000, Ruixi Biotech, China) was loaded to FeAuNPs to prepare ICG‐P‐MMCNPs and ICG‐MMCNPs through Au‐S bonds as described above. After tail‐vein injections of 4 mg*·*mL^−1^ ICG‐P‐MMCNPs at the dose of 3 µL*·*g^−1^, its distribution in vivo was monitored using a Xenogen IVIS Kinetic imaging system. To gain a clearer understanding of the tumor and organ distribution of P‐MMCNPs, we euthanized the mice 96 h after intravenous injection of P‐MMCNPs, perfused vital organs with PBS, and subsequently isolated the organs for fluorescence imaging. The distribution of P‐MMCNPs in tumor sections was also detected using a laser confocal microscope (Olympus, FV31s‐sw, Japan).

#### 4.1.14 MRI/CT Imaging

MRI/CT imaging properties of P‐MMCNPs in vitro were acquired using the MiniMR‐60 system (NIUMAG, China) and Micro‐CT (SKYSCAN 1276, Bruker, Germany), respectively. To investigate P‐MMCNPs targeting accumulation in tumor tissue, MRI/CT studies were performed in PC3^PSMA+^‐luc subcutaneous tumor‐bearing mice. Each mouse was anesthetized with isoflurane and placed in an animal coil 2 h post‐intravenous injection of 4 mg*·*mL^−1^ P‐MMCNPs. Parameter setting: (1) T1‐weighted imaging (T1WI): Time of repetition (TR) = 400 ms and time of echo delay (TE) = 20 ms; (2) T2‐weighted imaging (T2WI): TR = 1500 ms and TE = 80 ms; (3) CT: Scanning voltage = 45 kV and scanning current = 200 µA; (4) Slice thickness = 2 mm and slice gap = 0.5 mm. Signal intensity was measured using ImageJ software.

#### 4.1.15 Detection of Circulatory Half‐Life

To investigate the circulatory half‐life, 4 mg*·*mL^−1^ FeAuNPs@DM1, P‐MMCNPs@DM1, or MMCNPs@DM1 were injected into normal BALB/c nude mice intravenously. 20 µL blood samples were collected from the tail at a specific time after injection. For the detection of DM1 in serum, the serum samples underwent a pretreatment process. An equal volume of acetonitrile was added to the serum, followed by vortexing for 2 min. The mixture was then centrifuged at room temperature at 14,000 g for 5 min. The resulting supernatant was collected and subjected to HPLC analysis. The flow rate was set to 1 mL*·*min^−1^, and the column temperature was maintained at 35°C. DM1 was detected using a UV detector at 290–300 nm, with a retention time of 4.64 min.

#### 4.1.16 IC50 Determination

To evaluate DM1‐induced cytotoxicity, we successfully formulated P‐MMCNPs@DM1 adhering to the optimal ratio identified through DM1 loading studies (Supplementary Table S1), that is, FeAuNPs: DM1: Macrophage membrane = 200:10:400. Following this, we performed IC50 assays through serial dilutions of P‐MMCNPs@DM1, with gradients calculated from theoretical concentrations of DM1. P‐MMCNPs@DM1 was incubated with PC3 cells for 24 h. Then, the plate was washed with PBS, and cell viability was measured with a CCK‐8 assay (Beyotime Biotechnology, C0038, China) according to the user manual. Results were recorded using a microplate reader (Infinite 200, Tecan, Switzerland) at 450 nm. IC50 was derived from the variable slope model. Regarding P‐MMCNPs‐mediated PTT, the laser irradiation parameters were set at 0.5 W·cm^−2^ for a duration of 5 min.

#### 4.1.17 Tubulin Staining

1 × 10^5^ PC3^PSMA+^ cells were seeded into confocal dishes for overnight incubation. Subsequently, the medium was replaced with fresh medium containing P‐MMCNPs@DM1 (200  µg*·*mL^−1^ P‐MMCNPs loaded with 1.2 µg*·*mL^−1^ DM1). After 24 h incubation, cells were washed with PBS, fixed with 4% paraformaldehyde, and blocked with primary antibody dilution buffer. After incubating with α‐tubulin rabbit polyclonal antibody (Proteintech, 11224‐1‐AP, China) overnight at 4°C, dishes were incubated with Alexa Fluor 488‐conjugated donkey anti‐rabbit IgG (Thermo Fisher, R37118, USA) for 2 h at room temperature. Digital images were obtained using a Zeiss laser confocal microscope (LSM 800, Germany). ImageJ software was adopted for quantification.

#### 4.1.18 Combined Anti‐Tumor Effects in Vivo

PC3^PSMA+^‐luc xenograft mice were constructed and randomly divided into five groups as mentioned above. For the treatment group, 4 mg*·*mL^−1^ P‐MMCNPs or P‐MMCNPs@DM1 (loaded with 24 µg*·*mL^−1^ DM1) were tail‐vein‐injected every 3 days at the dose of 3 µL*·*g^−1^, so that their blood concentration could reach the calculated IC50 in vitro (blood volume of mice was reported as 58.5 µL*·*g^−1^). 24 h later, animals in the PTT group were irradiated with an 808 nm laser for 5 min (MDL‐III‐808, Changfu Technology, China). The specific laser parameters are as follows: spot diameter of approximately 6 mm; wavelength stability of ± 3  nm; power density of 0.5 W*·*cm^−2^; irradiation distance of 2 cm. Tumor growth was monitored with bioluminescent imaging.

#### 4.1.19 Ki67 and TUNEL Staining

After 4 weeks of treatment, the tumors of subcutaneous tumor‐bearing mice were resected for morphological staining. Ki67 (Beyotime, AF1738, China) and TUNEL (Beyotime, C1089, China) staining were performed as previously reported. Digital images were obtained using a laser confocal microscope (Olympus, FV31s‐sw, Japan). The percentage of apoptotic and proliferating cells was calculated by the researchers, who were blind to the experimental design.

#### 4.1.20 Tibial Micro‐Structure Analysis

After the treatment experiment, tumor‐metastatic tibias were collected for micro‐CT examination. The coronal plane of the tibia was placed parallel to the examination bed in the test chamber. The parameter setting was scanning voltage = 45 kV; scanning current = 200 µA; spatial resolution = 8 µm; and bone mineral density > 80. NRecon software and CTvox software were adopted for 3D reconstruction and rendering, respectively. Data Viewer software was used for image visualization. CTan software was employed for the region of interest selection and bone metrological analysis.

#### 4.1.21 Macrophage‐Related Section Staining

4‐µm‐thick tissue sections were applied to antigen retrieval in citrate solution. Being blocked with goat serum for 1 h at room temperature, slices were incubated with primary antibodies: anti‐CD68 (Abcam, ab53444, USA), anti‐iNOS (Abcam, ab178945, USA), anti‐CD206 (Abcam, ab64693, USA), anti‐E‐cadherin (ECAD) (Proteintech, 20874‐1‐AP, China), anti‐vimentin (Proteintech, 10366‐1‐AP, China), anti‐vascular endothelial growth factor receptor 2 (VEGFR2) (Proteintech, 26415‐1‐AP, China), and anti‐CD105 (Proteintech, 10862‐1‐AP, China) in a humidified box overnight at 4°C. Slices were then incubated with Alexa Fluor 488 (Abcam, ab150157, USA) and Alexa Fluor 594 (Abcam, ab150080, USA) conjugated secondary antibodies for 2 h at room temperature. Nuclei were stained with DAPI. Digital images were acquired using a laser confocal microscope (Olympus, FV31s‐sw, Japan). Quantitative analysis was performed using ImageJ software.

#### 4.1.22 ELISA Assay for Macrophage‐Related Cytokines

The resected tumors were weighed and homogenized in lysis buffer supplemented with proteinase inhibitor using a homogenizer device (Leica, Germany). The supernatant was collected after centrifugation at 14,000 g for 20 min at 4°C. ELISA kits (Proteintech, China) were employed for cytokine detection according to the manufacturer's instructions. The absorbance was measured by using a microplate reader at 450 nm (Infinite 200, Tecan, Switzerland).

#### 4.1.23 qRT‐PCR Assay

The resected tumors were employed for VEGFC expression level detection. The experimental protocol of qRT‐PCR was the same as previously reported. The sequences of the VEGFC primer were GAGGTCAAGGCTTTTGAAGGC (F) and CTGTCCTGGTATTGAGGGTGG (R).

#### 4.1.24 Organ‐Toxicity Evaluation of P‐MMCNPs

After two consecutive weeks of P‐MMCNPs or P‐MMCNPs@DM1 injection, normal C57 mice were sacrificed by neck dislocation, and blood samples were taken from the left ventricle of the heart. 200 µL serum was collected from blood after centrifugation at 3000 rpm for 10 min at 4°C. Serum biochemical markers were detected by commercial kits and a multifunctional biochemistry analyzer (Olympus, AU600, Japan) according to the manufacturer's instructions. Meanwhile, major organs of mice injected with P‐MMCNPs were resected and fixed with 4% paraformaldehyde. Fetal mice were collected from the pregnant C57 mice after two‐week injections of P‐MMCNPs. After being embedded in paraffin, 4‐µm‐thick slices were obtained for H&E staining. Digital images were acquired using an inverted microscope (Nikon, TE2000‐S, Japan). Additionally, fetal mice were stained with alizarin red/alixin blue solution (Servicebio, G1038 and G1027, China) according to the manufacturer's instructions after being fixed with 95% ethanol. Photographs were taken with a stereomicroscope in 50% glycerol.

#### 4.1.25 Statistical Analysis

Student *t*‐test (two‐tailed), one‐way analysis of variance (ANOVA), and repeated‐measures ANOVA were used for data analysis. Data were presented as mean ± SD from at least triplicate measurements. Statistical analyses were performed using SPSS 20.0 (SPSS, USA) or R software (https://www.r‐project.org). *P* < 0.05 was considered statistically significant.

## Author Contributions

K. Zhang, B. Gao, J. Wang, Y. Li, Y. Zou, D. Han, H. Bian and W. Qin designed experiments and conceived the manuscript. K. Zhang, B. Gao, J. Wang, Y. Li, C. Xu, F. Yang, S. Liu, H. Li, C. Zhang, X. Meng and Z. Shi performed most of the experiments. R. Zhang, Z. Wang, W. Wen and Q. Zhang assisted in manuscript writing. Y. Zou, D. Han, H. Bian and W. Qin directed the project.

## Ethics Statement

Animal experiments reported in this study were reviewed and approved by the Institutional Animal Care and Use Committee of the Fourth Military Medical University (approval number: 20201207). All applicable institutional guidelines for the care and use of animals were followed.

## Conflicts of Interest

The authors declare no conflicts of interest.

## Supporting information




**Supporting File 1**: exp270145‐sup‐0001‐SuppMat.docx.

## Data Availability

There was no disagreement between any authors regarding the final version of the manuscript. This article presents comprehensive information to substantiate its findings. The corresponding authors can provide the data upon reasonable request.

## References

[1] R. L. Siegel , K. D. Miller , H. E. Fuchs , and A. Jemal , “Prostate Cancer,” CA: A Cancer Journal for Clinicians 72, no. 1 (2022): 7–33. 35020204 10.3322/caac.21708

[2] J. E. Damber and G. Aus , “Prostate Cancer,” Lancet 371, no. 9625 (2008): 1710–1721, 10.1016/S0140-6736(08)60729-1. 18486743

[3] I. de Lázaro and D. J. Mooney , "Obstacles and Opportunities in a Decade of Biomedical Nanoscience." Nature Materials 20, no. 11 (2021): 1469–1479. 34226688 10.1038/s41563-021-01047-7

[4] R. Cai and C. Chen , “The Crown and the Scepter: Roles of the Protein Corona in Nanomedicine,” Advanced Materials 31, no. 45 (2019): e1805740, 10.1002/adma.201805740. 30589115

[5] X. Wang , X. Zhong , J. Li , Z. Liu , and L. Cheng , “Inorganic Nanomaterials With Rapid Clearance for Biomedical Applications,” Chemical Society Reviews 50, no. 15 (2021): 8669–8742, 10.1039/D0CS00461H. 34156040

[6] X. Zhen , P. Cheng , and K. Pu , “Recent Advances in Cell Membrane–Camouflaged Nanoparticles for Cancer Phototherapy,” Small 15, no. 1 (2019): e1804105, 10.1002/smll.201804105. 30457701

[7] C. Qiao , X. Wang , G. Liu , et al., “Erythrocyte Membrane Camouflaged Metal–Organic Framework Nanodrugs for Remodeled Tumor Microenvironment and Enhanced Tumor Chemotherapy,” Advanced Functional Materials 32, no. 10 (2022): 2107791, 10.1002/adfm.202107791.

[8] Y. Zhai , J. Wang , T. Lang , et al., “T Lymphocyte Membrane‐decorated Epigenetic Nanoinducer of Interferons for Cancer Immunotherapy,” Nature Nanotechnology 16, no. 11 (2021): 1271–1280, 10.1038/s41565-021-00972-7. 34580467

[9] B. Bahmani , H. Gong , B. T. Luk , et al., “Intratumoral Immunotherapy Using Platelet‐Cloaked Nanoparticles Enhances Antitumor Immunity in Solid Tumors,” Nature Communications 12 (2021): 1999, 10.1038/s41467-021-22311-z. PMC8012593 33790276

[10] Y. Jiang , N. Krishnan , J. Zhou , et al., “Engineered Cell‐Membrane‐Coated Nanoparticles Directly Present Tumor Antigens to Promote Anticancer Immunity,” Advanced Materials 32, no. 30 (2020): e2001808, 10.1002/adma.202001808. 32538494 PMC7669572

[11] H. H. Wu , Y. Zhou , Y. Tabata , and J. Q. Gao , “Mesenchymal Stem Cell‐Based Drug Delivery Strategy: From Cells to Biomimetic,” Journal Control Release 294 (2019): 102–113, 10.1016/j.jconrel.2018.12.019. 30553849

[12] P. S. Coburn , F. C. Miller , A. L. LaGrow , et al., “Disarming Pore‐Forming Toxins With Biomimetic Nanosponges in Intraocular Infections,” mSphere 4, no. 3 (2019): e00262‐19, 10.1128/mSphere.00262-19. PMC6520441 31092603

[13] C. Chen , M. Song , Y. Du , et al., “Tumor‐Associated‐Macrophage‐Membrane‐Coated Nanoparticles for Improved Photodynamic Immunotherapy,” Nano Letters 21, no. 13 (2021): 5522–5531, 10.1021/acs.nanolett.1c00818. 34133181

[14] L. Liu , X. Bai , M. V. Martikainen , et al., “Cell Membrane Coating Integrity Affects the Internalization Mechanism of Biomimetic Nanoparticles,” Nature Communications 12 (2021): 5726, 10.1038/s41467-021-26052-x. PMC8484581 34593813

[15] J. Mosquera , I. García , and L. M. Liz‐Marzán , “Cellular Uptake of Nanoparticles versus Small Molecules: A Matter of Size,” Accounts of Chemical Research 51, no. 9 (2018): 2305–2313, 10.1021/acs.accounts.8b00292. 30156826

[16] U. Haberkorn , M. Eder , K. Kopka , J. W. Babich , and M. Eisenhut , “New Strategies in Prostate Cancer: Prostate‐Specific Membrane Antigen (PSMA) Ligands for Diagnosis and Therapy,” Clinical Cancer Research 22, no. 1 (2016): 9–15, 10.1158/1078-0432.CCR-15-0820. 26728408

[17] D. Han , J. Wu , Y. Han , et al., “A Novel Anti‐PSMA Human scFv Has the Potential to be Used as a Diagnostic Tool in Prostate Cancer,” Oncotarget 7, no. 37 (2016): 59471–59481, 10.18632/oncotarget.10697. 27448970 PMC5312325

[18] S. J. Shi , L. J. Wang , D. H. Han , et al., “Therapeutic Effects of Human Monoclonal PSMA Antibody‐Mediated TRIM24 siRNA Delivery in PSMA‐Positive Castration‐Resistant Prostate Cancer,” Theranostics 9, no. 5 (2019): 1247–1263, 10.7150/thno.29884. 30867828 PMC6401511

[19] J. Wu , D. Han , S. Shi , et al., “A Novel Fully Human Antibody Targeting Extracellular Domain of PSMA Inhibits Tumor Growth in Prostate Cancer,” Molecular Cancer Therapeutics 18, no. 7 (2019): 1289–1301, 10.1158/1535-7163.MCT-18-1078. 31048359

[20] Z. Xu , Y. Hou , and S. Sun , “Magnetic Core/Shell Fe_3_O_4_/Au and Fe_3_O_4_/Au/Ag Nanoparticles With Tunable Plasmonic Properties,” Journal of the American Chemical Society 129, no. 28 (2007): 8698–8699, 10.1021/ja073057v. 17590000

[21] C. T. Huang , X. Guo , C. Bařinka , et al., “Development of 5D3‐DM1: A Novel Anti‐Prostate‐Specific Membrane Antigen Antibody‐Drug Conjugate for PSMA‐Positive Prostate Cancer Therapy,” Molecular Pharmacology 17, no. 9 (2020): 3392–3402, 10.1021/acs.molpharmaceut.0c00457. PMC7957835 32803984

[22] P. Zhang , Y. Qiao , L. Zhu , et al., “Nanoprobe Based on Biominerals in Protein Corona for Dual‐Modality MR Imaging and Therapy of Tumors,” ACS Nano 17, no. 2 (2023): 184–196, 10.1021/acsnano.2c05917. 36525358

[23] X. Li , Y. Li , C. Yu , et al., “ROS‐Responsive Janus Au/Mesoporous Silica Core/Shell Nanoparticles for Drug Delivery and Long‐Term CT Imaging Tracking of MSCs in Pulmonary Fibrosis Treatment,” ACS Nano 17, no. 7 (2023): 6387–6399, 10.1021/acsnano.2c11112. 36946383

[24] B. Wan , Q. Bao , and D. Burgess , “Long‐Acting PLGA Microspheres: Advances in Excipient and Product Analysis Toward Improved Product Understanding,” Advanced Drug Delivery Reviews 198 (2023): 114857, 10.1016/j.addr.2023.114857. 37149041

[25] Y. Xia , L. Rao , H. Yao , Z. Wang , P. Ning , and X. Chen , “Engineering Macrophages for Cancer Immunotherapy and Drug Delivery,” Advanced Materials 32, no. 51 (2020): e2002054, 10.1002/adma.202002054. 32856350

[26] P. Zhang , L. Zhang , Z. Qin , et al., “Genetically Engineered Liposome‐like Nanovesicles as Active Targeted Delivery Platform,” Advanced Materials 30, no. 8 (2018): e1705350. 10.1002/adma.201705350 29280210

[27] A. M. Satar , F. A. Othman , and S. C. Tan , “Biomaterial Application Strategies to Enhance Stem Cell‐Based Therapy for Ischemic Stroke,” World Journal Stem Cells 14, no. 12 (2022): 851–867, 10.4252/wjsc.v14.i12.851. PMC9813837 36619694

[28] L. Liu , H. He , and J. Liu , “Advances on Non‐Genetic Cell Membrane Engineering for Biomedical Applications,” Polymers 11, no. 11 (2019): 2017. 31817418 10.3390/polym11122017 PMC6961000

[29] L. Woythe , D. Porciani , T. Harzing , S. van Veen , D. H. Burke , and L. Albertazzi , “Valency and Affinity Control of Aptamer‐conjugated Nanoparticles for Selective Cancer Cell Targeting,” Journal Control Release 355 (2023): 228–237, 10.1016/j.jconrel.2023.01.008. 36642253

[30] A. Fakhari , A. Baoum , T. J. Siahaan , K. B. Le , and C. Berkland , “Controlling Ligand Surface Density Optimizes Nanoparticle Binding to ICAM‐1,” Journal of Pharmaceutical Sciences 100, no. 3 (2011): 1045–1056, 10.1002/jps.22342. 20922813 PMC4245447

[31] B. Schäfer , E. Orbán , A. Borics , et al., “Preparation of Semisynthetic Lipoproteins With Fluorescent Cholesterol Anchor and Their Introduction to the Cell Membrane With Minimal Disruption of the Membrane,” Bioconjugate Chemistry 24, no. 10 (2013): 1684–1697, 10.1021/bc4002135. 24020959

[32] M. E. Aikins , C. Xu , and J. J. Moon , “Engineered Nanoparticles for Cancer Vaccination and Immunotherapy,” Accounts of Chemical Research 53, no. 10 (2020): 2094–2105, 10.1021/acs.accounts.0c00456. 33017150 PMC7871038

[33] P. Lv , X. Liu , X. Chen , et al., “Genetically Engineered Cell Membrane Nanovesicles for Oncolytic Adenovirus Delivery: A Versatile Platform for Cancer Virotherapy,” Nano Letters 19, no. 5 (2019): 2993–3001, 10.1021/acs.nanolett.9b00145. 30964695

[34] C. Hu , C. A. Leche 2nd , A. Kiyatkin , et al., “Glioblastoma Mutations Alter EGFR Dimer Structure to Prevent Ligand Bias,” Nature 602, no. 7897 (2022): 518–522, 10.1038/s41586-021-04393-3. 35140400 PMC8857055

[35] J. Zhang , D. Ji , L. Cai , et al., “First‐in‐human HER2‐targeted Bispecific Antibody KN026 for the Treatment of Patients With HER2‐positive Metastatic Breast Cancer: Results From a Phase I Study,” Clinical Cancer Research 28, no. 4 (2022): 618–628, 10.1158/1078-0432.CCR-21-2827. 34844975

[36] X. Wu , H. Luo , B. Shi , et al., “Combined Antitumor Effects of Sorafenib and GPC3‐CAR T Cells in Mouse Models of Hepatocellular Carcinoma,” Molecular Pharmacology 27, no. 8 (2019): 1483–1494, 10.1016/j.ymthe.2019.04.020. PMC6697347 31078430

[37] R. H. Fang , W. Gao , and L. Zhang , “Targeting Drugs to Tumours Using Cell Membrane‐coated Nanoparticles,” Nature Reviews Clinical Oncology 20, no. 1 (2023): 33–48, 10.1038/s41571-022-00699-x. 36307534

[38] R. B. Berish , A. N. Ali , P. G. Telmer , J. A. Ronald , and H. S. Leong , “Translational Models of Prostate Cancer Bone Metastasis,” Nature Reviews Urology 15, no. 7 (2018): 403–421, 10.1038/s41585-018-0020-2. 29769644

[39] J. M. Cassady , K. K. Chan , H. G. Floss , and E. Leistner , “Recent Developments in the Maytansinoid Antitumor Agents,” Chemical and Pharmaceutical Bulletin 52, no. 1 (2004): 1–26, 10.1248/cpb.52.1. 14709862

[40] B. Luk , Y. Jiang , J. Copp , et al., “Biomimetic Targeting of Nanoparticles to Immune Cell Subsets via Cognate Antigen Interactions,” Molecular Pharmacology 15, no. 7 (2018): 3723–3728, 10.1021/acs.molpharmaceut.8b00074. PMC6123265 29533668

[41] J. A. Copp , R. H. Fang , B. T. Luk , C. M. J. Hu , and L. Zhang , “Clearance of Pathological Antibodies Using Biomimetic Nanoparticles,” Proceedings National Academy of Science USA 111, no. 37 (2014): 13481–13486, 10.1073/pnas.1412420111. PMC4169917 25197051

[42] S. H. Park , “Diagnosis and Treatment of Autoimmune Hemolytic Anemia: Classic Approach and Recent Advances,” Blood Research 51, no. 2 (2016): 69, 10.5045/br.2016.51.2.69. 27382547 PMC4931937

[43] X. Wei , J. Gao , R. H. Fang , et al., “Nanoparticles Camouflaged in Platelet Membrane Coating as an Antibody Decoy for the Treatment of Immune Thrombocytopenia,” Biomaterials 111 (2016): 116–123, 10.1016/j.biomaterials.2016.10.003. 27728811 PMC5082416

[44] D. B. Cines , J. B. Bussel , H. A. Liebman , and E. T. Luning Prak , “The ITP Syndrome: Pathogenic and Clinical Diversity,” Blood 113, no. 26 (2009): 6511–6521, 10.1182/blood-2009-01-129155. 19395674 PMC2710913

[45] Y. Jiang , D. Nie , Z. Hu , et al., “Macrophage‐Derived Nanosponges Adsorb Cytokines and Modulate Macrophage Polarization for Renal Cell Carcinoma Immunotherapy,” Advanced Healthcare Materials 13, no. 14 (2024): e2400303, 10.1002/adhm.202400303. 38647150

[46] D. G. DeNardo and B. Ruffell , “Macrophages as Regulators of Tumour Immunity and Immunotherapy,” Nature Reviews Immunology 19, no. 6 (2019): 369–382, 10.1038/s41577-019-0127-6. PMC7339861 30718830

[47] Y. Yokozeki , A. Kawakubo , M. Miyagi , et al., “Reduced TGF‐ β Expression and CD206‐Positive Resident Macrophages in the Intervertebral Discs of Aged Mice,” BioMed Research International 2021 (2021): 7988320, 10.1155/2021/7988320. 34337052 PMC8289593

[48] J. Zhang , H. Li , Q. Wu , et al., “Tumoral NOX4 Recruits M2 Tumor‐associated Macrophages via ROS/PI3K Signaling‐dependent Various Cytokine Production to Promote NSCLC Growth,” Redox Biology 22 (2019): 101116, 10.1016/j.redox.2019.101116. 30769285 PMC6374999

[49] R. H. Fang , C. M. Hu , B. T. Luk , et al., “Cancer Cell Membrane‐Coated Nanoparticles for Anticancer Vaccination and Drug Delivery,” Nano Letters 14, no. 4 (2014): 2181–2188, 10.1021/nl500618u. 24673373 PMC3985711

[50] C. M. Hu , L. Zhang , S. Aryal , C. Cheung , R. H. Fang , and L. Zhang , “Erythrocyte Membrane‐Camouflaged Polymeric Nanoparticles as a Biomimetic Delivery Platform,” Proceedings of the National Academy of Science USA 108, no. 27 (2011): 10980–10985, 10.1073/pnas.1106634108. PMC3131364 21690347

