# Supplementary material for: PSMA‐Targeting Macrophage Membrane‐Coated Nanoparticles for Precision Diagnosis and Combination Therapy of Prostate Cancer
Source: Exploration (Beijing). 2026 Feb 22;6(2):20240393. doi: 10.1002/EXP.20240393 (PMC13094533; doi:10.1002/EXP.20240393)
Supplement: Supplementary file 1 — Supporting File 1: exp270145‐sup‐0001‐SuppMat.docx. [file EXP2-6-20240393-s001.docx]

**Supporting Information**

**
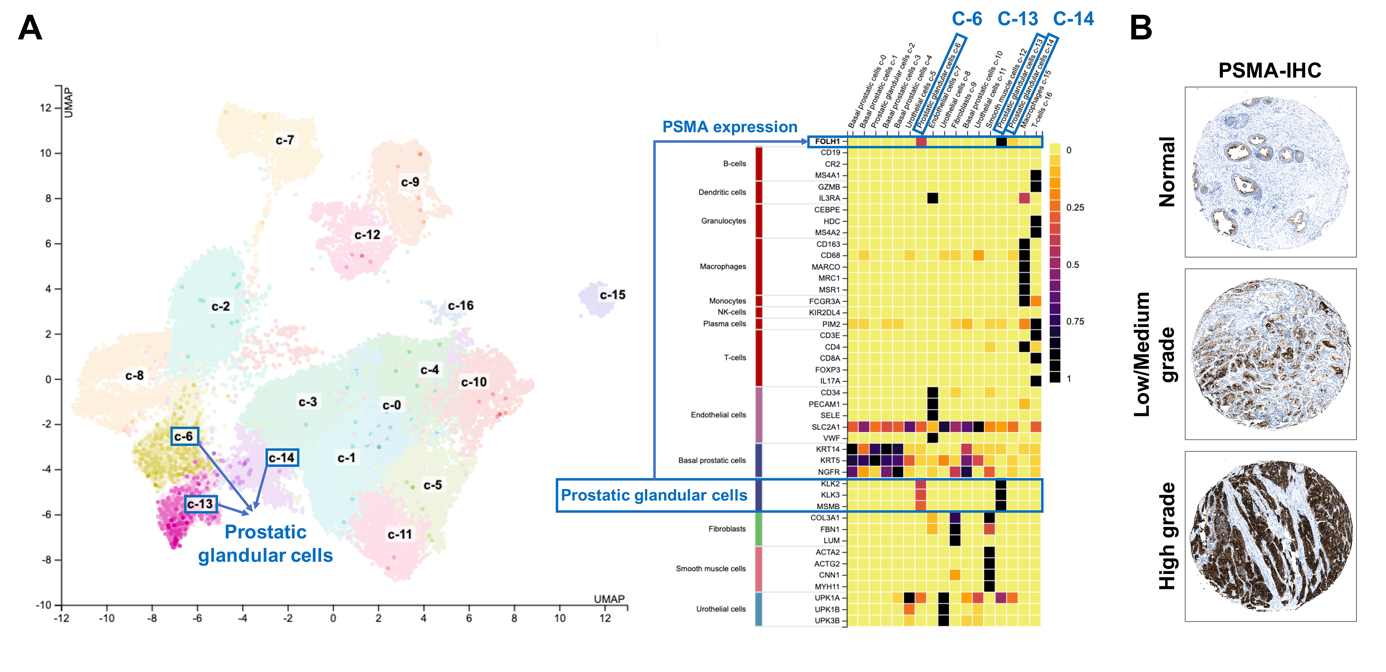
**

**Figure S1** Analysis of PSMA expression in prostate cancer (PCa). (A) Single-cell sequencing analysis of PSMA (*FOLH1*) expression in prostate. Prostatic glandular cells (i.e., c-6, c-13, and c-14) were marked with *KLK2, KLK3,* and *MSMB*. (B) Expression level of PSMA in PCa tissues retrieved from the HPA database. PSMA was marked with antibody HPA010593.

**
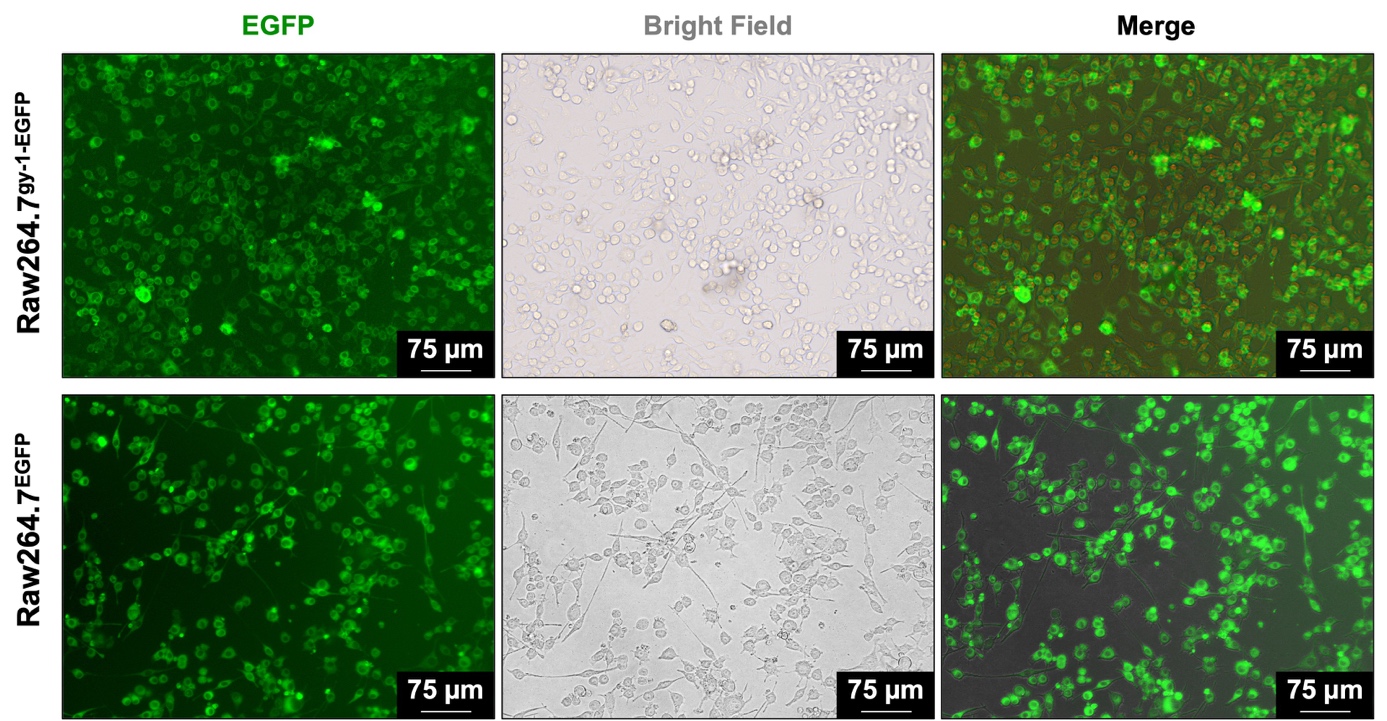
**

**Figure S2** Construction of engineered Raw246.7 cells transmembrane (TM)-expressing gy-1-EGFP fusion protein and EGFP fluorescent labeling. Scale bars: 75 μm.

**Figure S3** Expression of the fusion protein confirmed using Western blotting. (A) The gy-1-Hinge-TM-EGFP fusion protein was successfully expressed in Raw246.7^gy-1-EGFP^. (B) The fusion protein was present in P-MMCNPs and Raw246.7^gy-1-EGFP^ membranes.


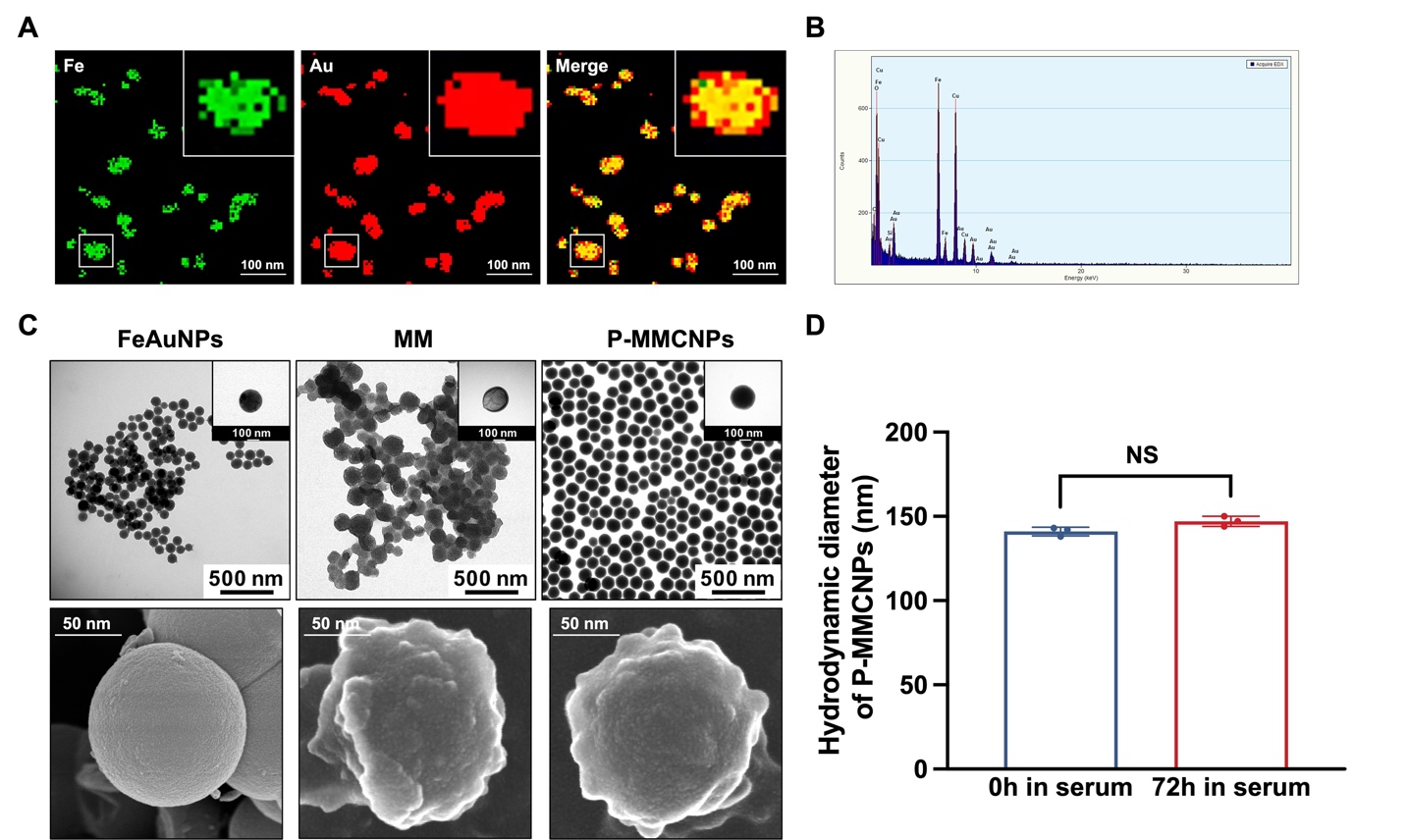


**Figure S4** Confirmation of core-shell structure of Fe_3_O_4_@Au nanoparticles (FeAuNPs). (A) Elemental mapping images of FeAuNPs. Scale bars: 100 nm. (B) Element composition and relative content of FeAuNPs. The Cu and Si peaks may arise from the carbon-coated copper grid commonly used in sample mounting. (C) The original TEM and SEM images of FeAuNPs, MM, and P-MMCNPs without pseudocolors. Scale bars, 500 nm, 100 nm, and 50 nm. (D) The hydrodynamic diameter of P-MMCNPs in serum analyzed using dynamic light scattering. NS, no significant.

**Figure S5** Drug release properties of FeAuNPs@DM1 and P-MMCNPs@DM1. (A) Drug release characteristics of FeAuNPs@DM1 and P-MMCNPs@DM1 in PBS. (B) GSH concentration within PCa cells. Normal cell lines 3T3 and Raw264.7 were used as controls. (C) GSH-triggered drug release characteristics of P-MMCNPs@DM1. Data were shown as mean ± SD from at least three independent experiments. ^****^*P* < 0.0001.


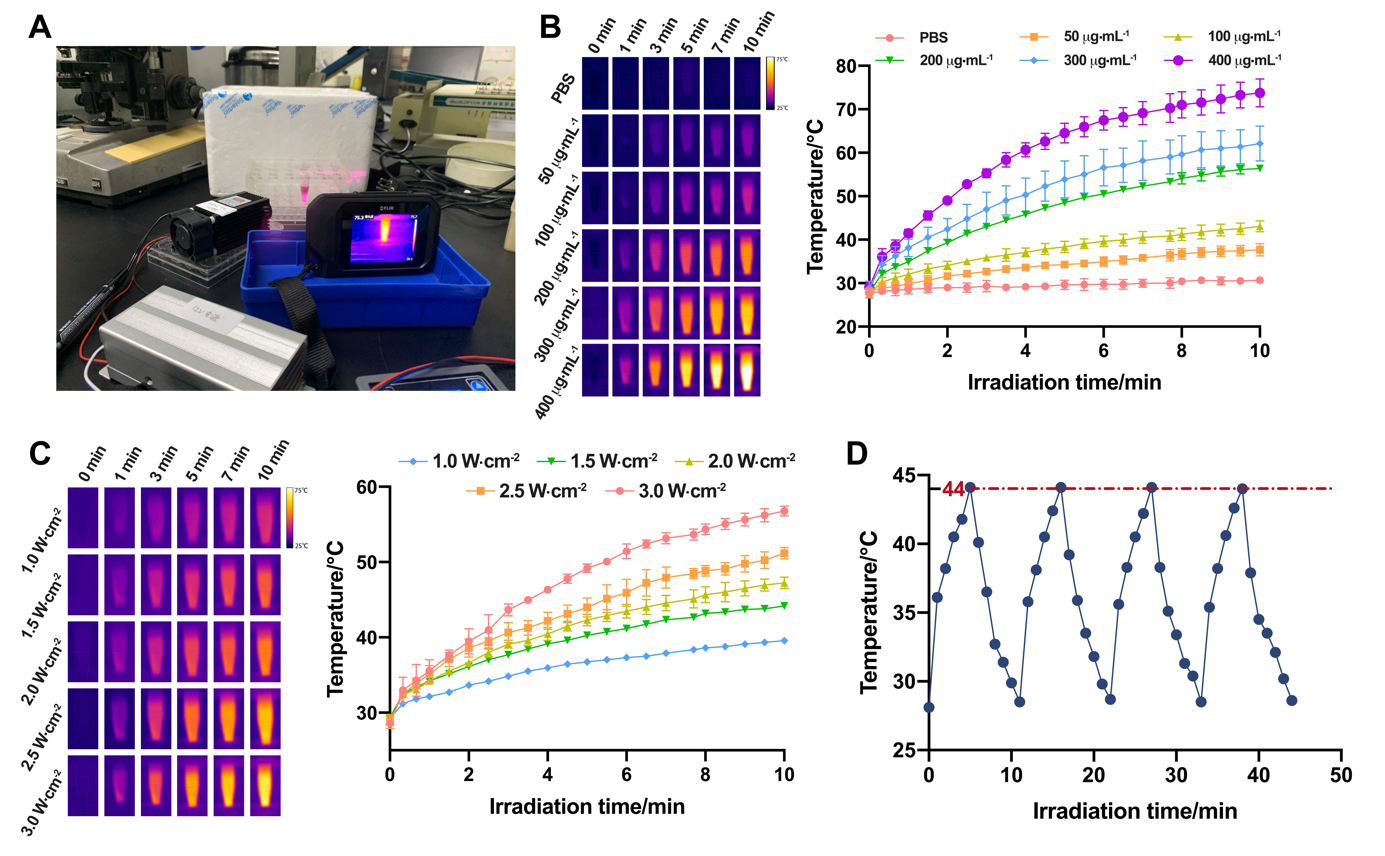


**Figure S6** Photothermal conversion capability of P-MMCNPs. (A) Experimental scene of P-MMCNPs photothermal conversion. (B) Concentration-dependent photothermal behavior of P-MMCNPs at a laser power density of 3 W*·*cm^–2^. (C) Laser power density-dependent photothermal behavior of P-MMCNPs (200 μg*·*mL^–1^). (D) Photothermal stability of P-MMCNPs. P-MMCNPs concentration was 200 μg*·*mL^–1^ and laser power density was 1.5 W*·*cm^–2^. Data were shown as mean ± SD from at least three independent experiments.

**Figure S7** MRI/CT capability of P-MMCNPs in vitro. (A) Dual-modality MRI capability of P-MMCNPs in vitro. (B) CT capability of P-MMCNPs in vitro.


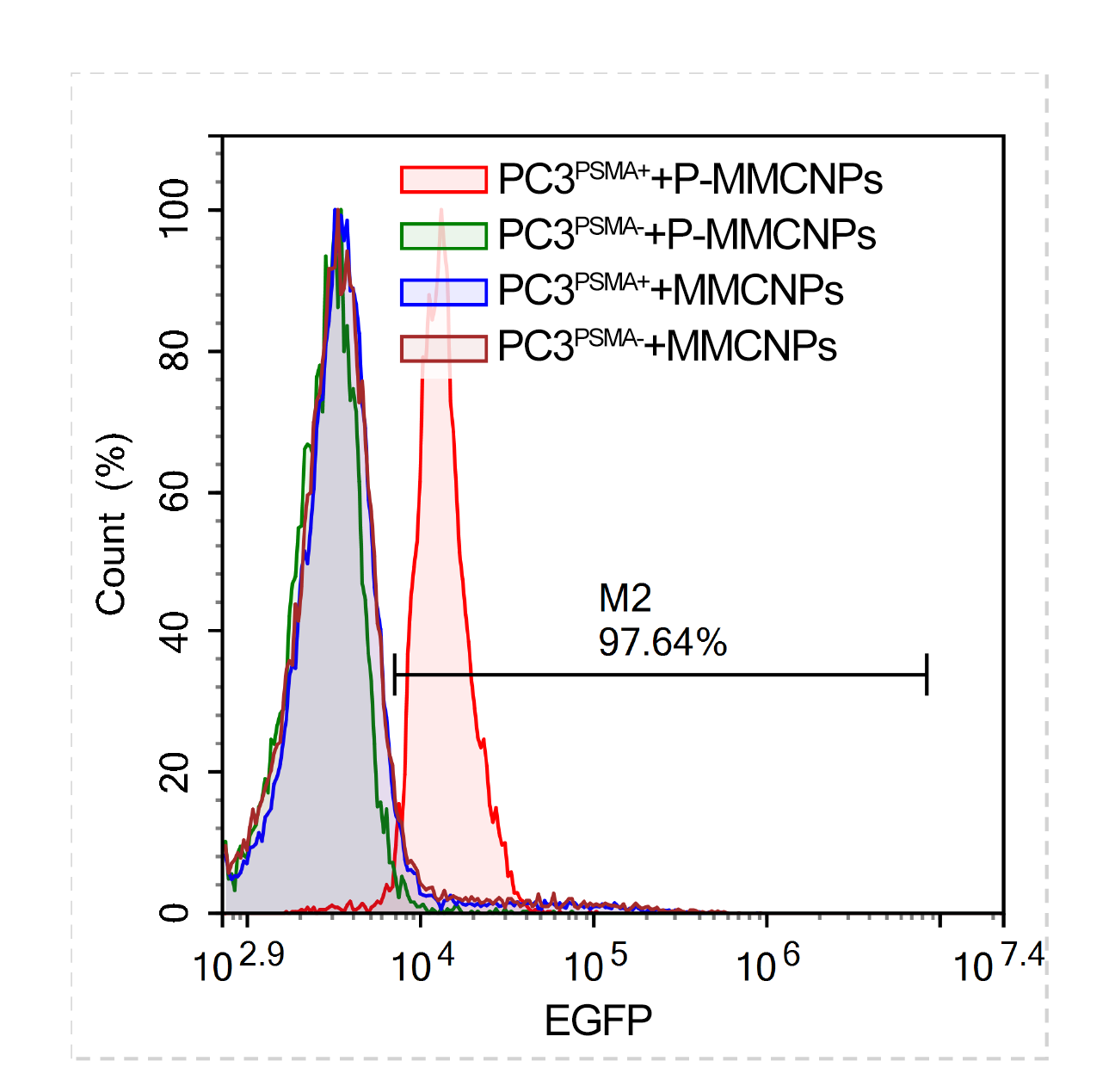


**Figure S8** P-MMCNPs with TM-expression of the gy-1-EGFP fusion protein specifically recognize PC3^PSMA+^ cells. MMCNPs were used as control.


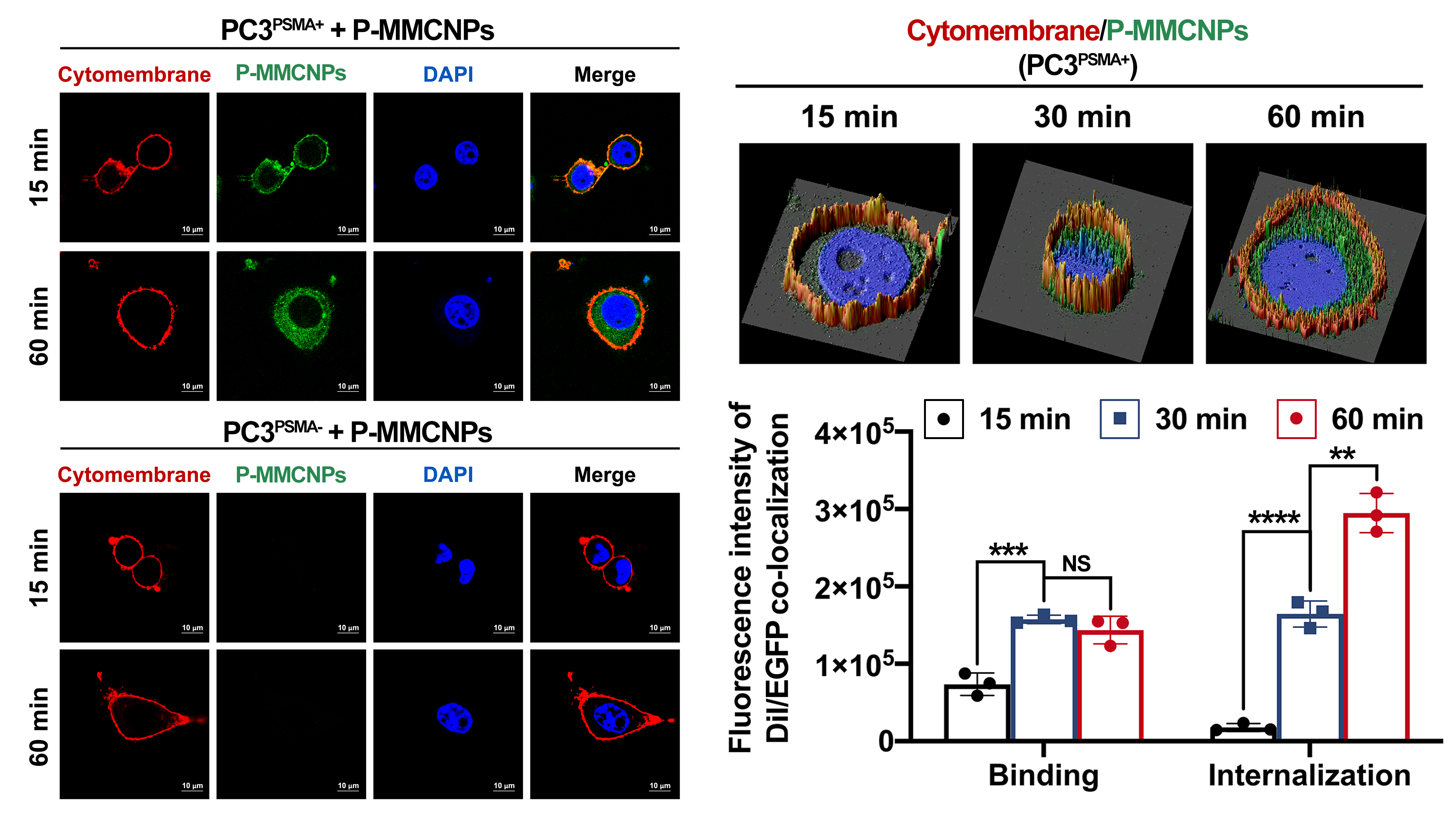


**Figure S9** Binding and internalization of P-MMCNPs to PC3^PSMA+^ cells. PC3^PSMA−^ cells were used as control. P-MMCNPs were labeled with gy-1-EGFP fusion protein (green). The cytomembrane was stained with Dil (red). Scale bars: 10 μm. Data were shown as mean ± SD. ^**^*P* < 0.01; ^***^*P* < 0.001; ^****^*P* < 0.0001; NS, no significant.


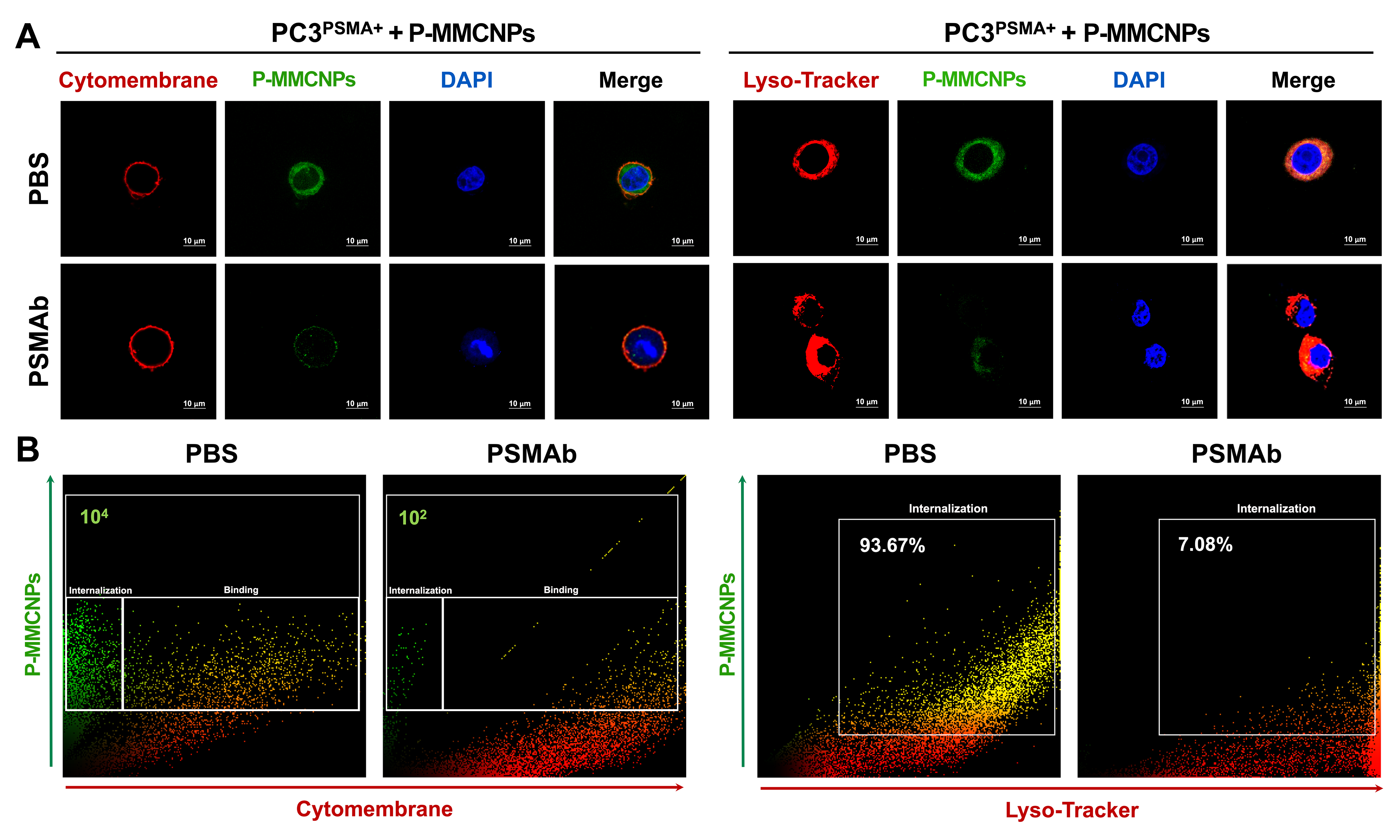


**Figure S10** Targeting effect of P-MMCNPs to PC3^PSMA+^ cells evaluated via PSMAb blocking. (A) TM-expressed gy-1-mediated binding and internalization of P-MMCNPs to PC3^PSMA+^ cells. (B) Quantification of fluorescent co-localization. PC3^PSMA+^ cells pre-treated with PSMAb having the same binding site as gy-1 were used as the blocking group. P-MMCNPs were labeled with gy-1-EGFP fusion protein (green). Cytomembrane and lysosome were stained with Dil and Lyso-Tracker (red), respectively. Scale bars: 10 μm.


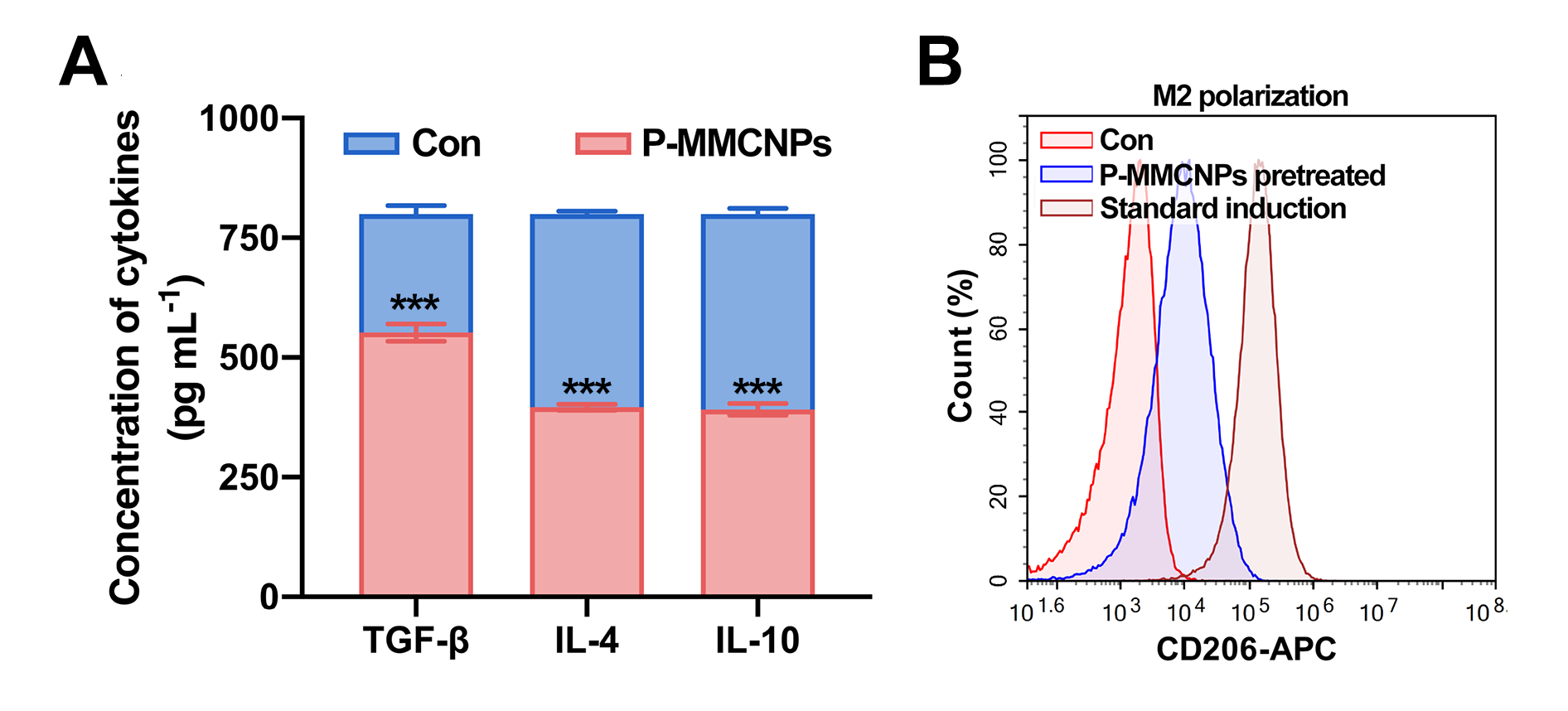


**Figure S11** Immunomodulatory potential of P-MMCNPs in vitro. (A) P-MMCNPs-mediated cytokine neutralization detected using ELISA. Untreated cytokines were used as the control. (B) P-MMCNPs-mediated M2 macrophage reprogramming detected using flow cytometry. Con: inactivated Raw264.7; Standard induction: 20 ng*·*mL^–1^ IL-4; P-MMCNPs pretreated: IL-4 for Raw264.7 stimulation was pre-treated with P-MMCNPs. ^***^*P* < 0.0001.


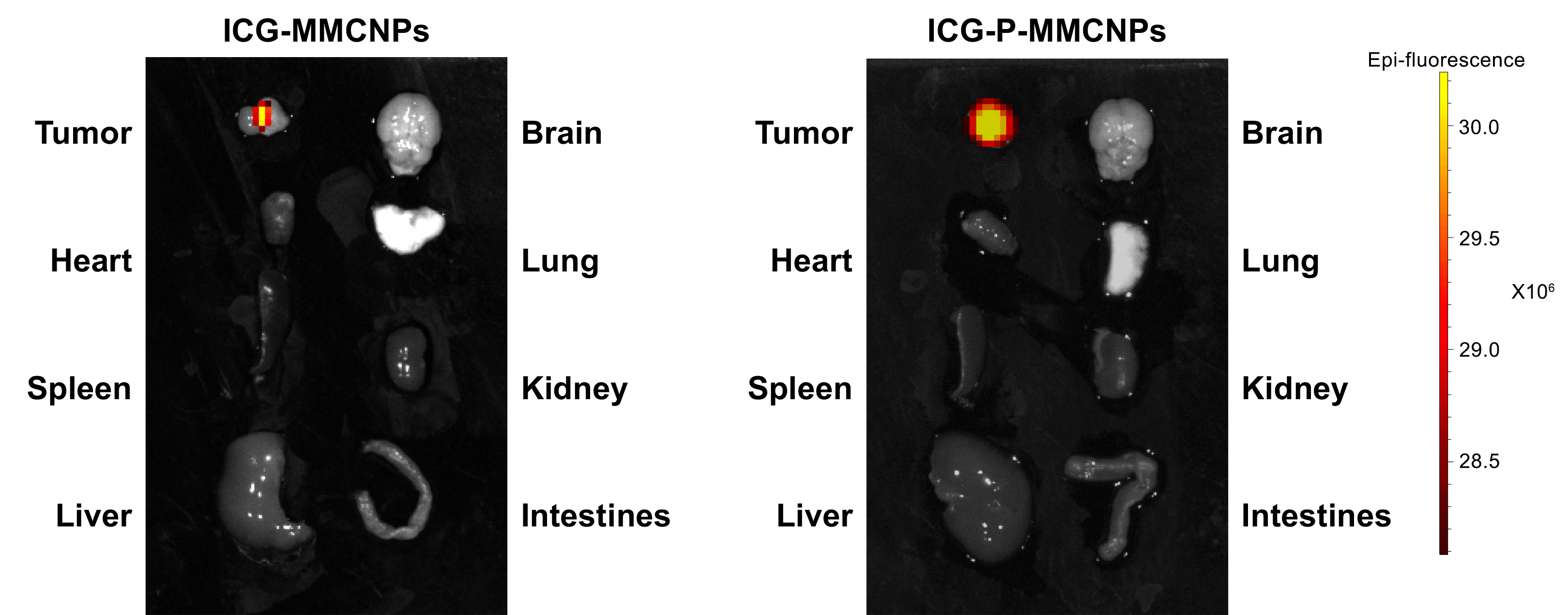


**Figure S12** In vivo distribution of ICG-P-MMCNPs and ICG-MMCNPs in PC3^PSMA+^ subcutaneous xenograft model at 96 hours post-intravenous injection.


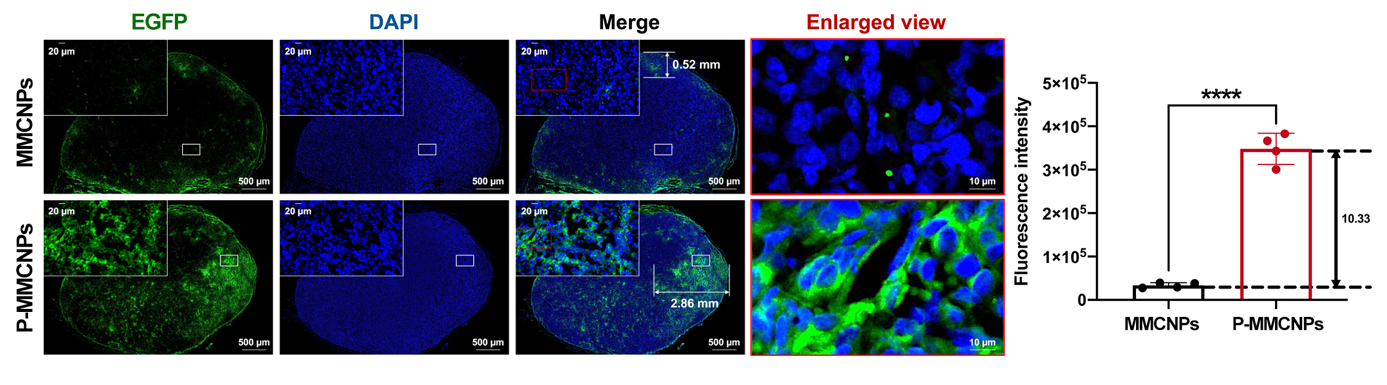


**Figure S13** Fluorescent localization of P-MMCNPs and MMCNPs in PC3^PSMA+^ subcutaneous xenograft model. P-MMCNPs were labeled with gy-1-EGFP fusion protein (green). MMCNPs were tracked with TM-expressed EGFP. Scale bars: 500 μm, 20 μm, and 10 μm. Data were shown as mean ± SD from three independent experiments. ^****^*P* < 0.0001.


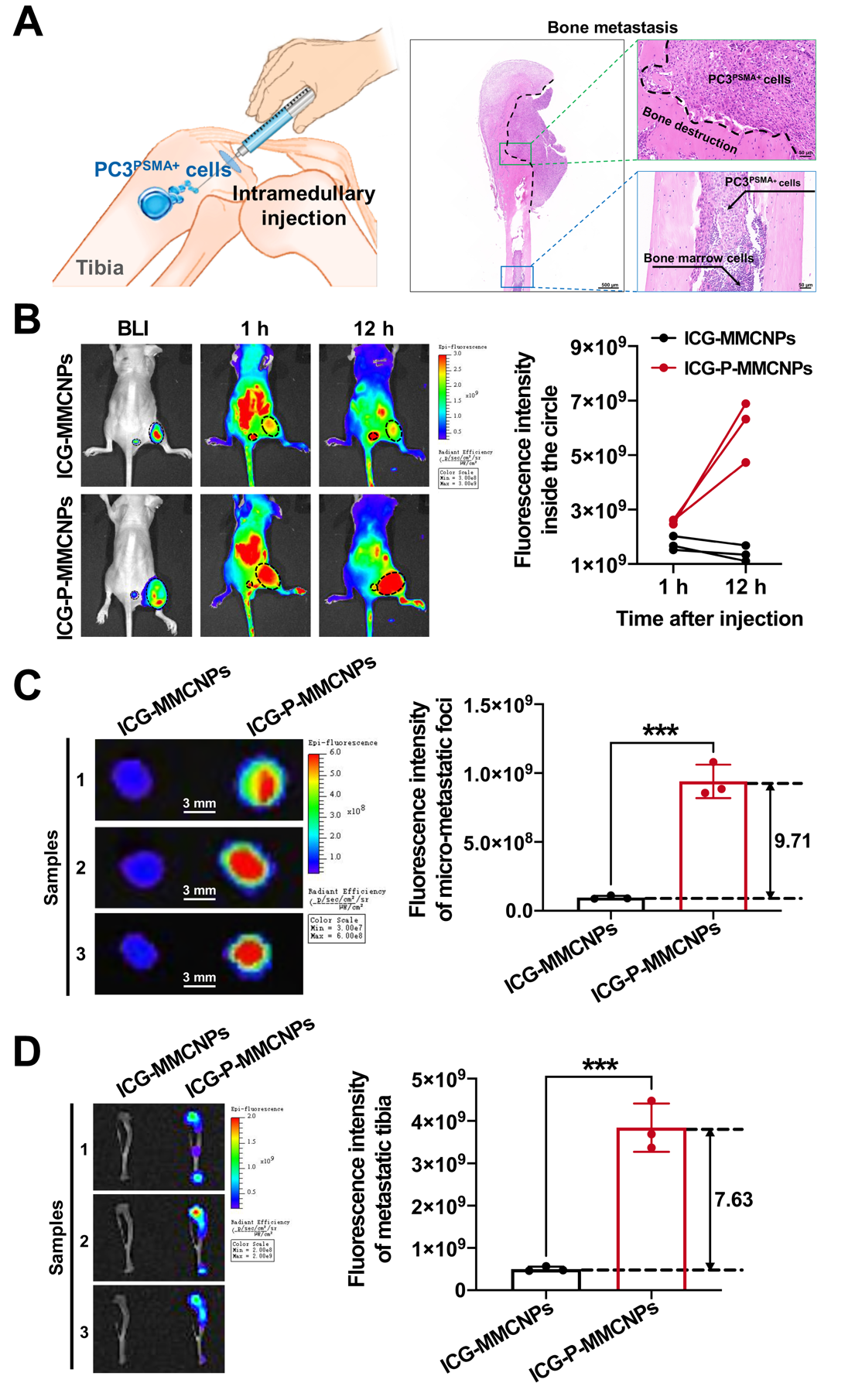


**Figure S14** Active tumor targeting of P-MMCNPs in PC3^PSMA+^ metastatic tumor-bearing mice. (A) H&E staining of the tibia with metastatic tumor confirming the successful construction of bone metastasis model. (B) Fluorescence signal quantification of tumor-metastatic tibia in vivo. (C and D) Quantitative analysis of ex vivo fluorescence signals in micro-metastatic foci (C) and tumor-metastatic tibia (D). Data were shown as mean ± SD. ^***^*P* < 0.001.

**Figure S15** CCK-8 assay showing the anti-tumor effects of FeAuNPs@DM1, MMCNPs@DM1, and P-MMCNPs@DM1 on the PC3^PSMA+^ cells. Untreated PCa cells were used as control. Data were analyzed using the student *t*-test (two-tailed). Data were shown as mean ± SD. ^**^*P* < 0.01; ^****^*P* < 0.0001


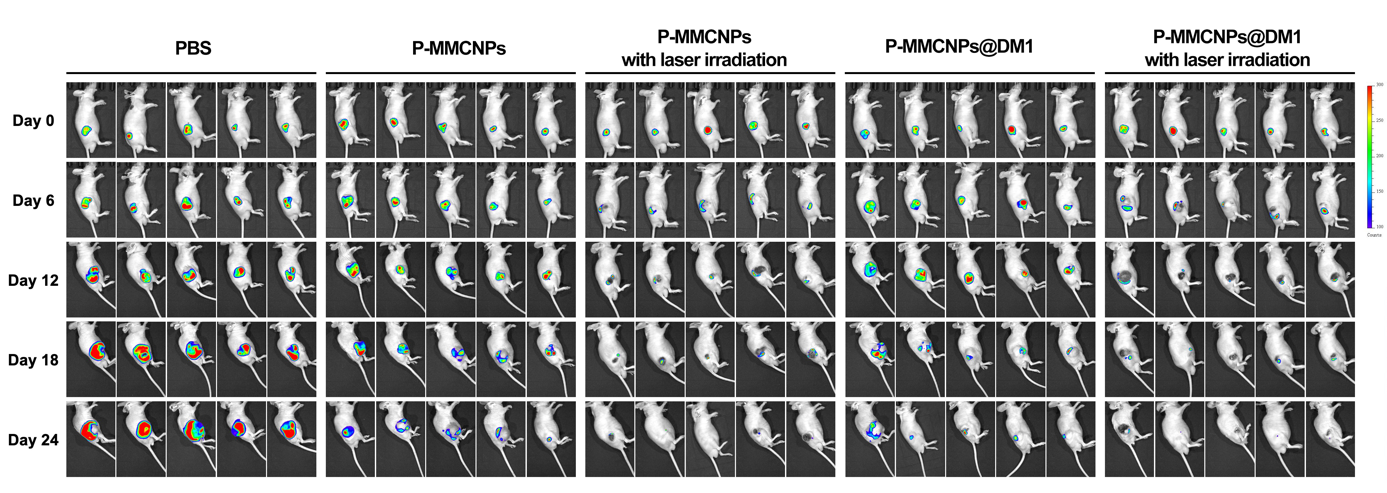


**Figure S16** Bioluminescent images showing P-MMCNPs active targeting-mediated combined anti-tumor effects in PC3^PSMA+^-luc subcutaneous xenograft model. Tumor-bearing mice injected with PBS were used as control. Data were shown as mean ± SD. n = 5 for each group.

**Figure S17** Comparison of therapeutic differences between FeAuNPs@DM1, MMCNPs@DM1, and P-MMCNPs@DM1 in the PC3^PSMA+^ subcutaneous xenograft model. (A) Schematic illustration to show the design of animal experiments in subcutaneous PCa model. (B) Growth curve of PC3^PSMA+^ tumors after indicated treatments. Data were analyzed using repeated-measures ANOVA. (C and D) Picture of isolated tumors (C) and tumor weights (D) after mice were sacrificed at endpoints. Data were analyzed using the student t-test (two-tailed). Data were shown as mean ± SD. n = 5 for each group. ^**^*P* < 0.01; ^***^*P* < 0.001; ^****^*P* < 0.0001.

**Figure S18** Fluorescent staining of osteoblasts and osteoclasts in the tibia with metastatic tumor. (A) OCN staining of osteoblasts. (B)TRAP staining of osteoclasts. Scale bars: 200 μm. Data were analyzed using the student t-test (two-tailed). Data were presented as mean ± SD from at least three independent experiments. ^****^*P* < 0.0001.

**Figure S19** Statistical analysis of CD68-positive cells in the immunofluorescence staining of tumor tissues. Data were analyzed using the student t-test (two-tailed). Tumor-bearing mice injected with PBS were used as control. NS, no significant.

**Figure S20** Flow cytometry of cell suspensions from tumor tissues after treatment with P-MMCNPs. (A) Increased proportion of M1 macrophages in cell suspensions of tumor tissue after treatment with P-MMCNPs. (B) Reduced proportion of M2 macrophages in cell suspensions of tumor tissue after treatment with P-MMCNPs. Data were analyzed using the student t-test (two-tailed). Tumor-bearing mice injected with PBS were used as control. ^*^*P* < 0.05; ^**^*P* < 0.01.


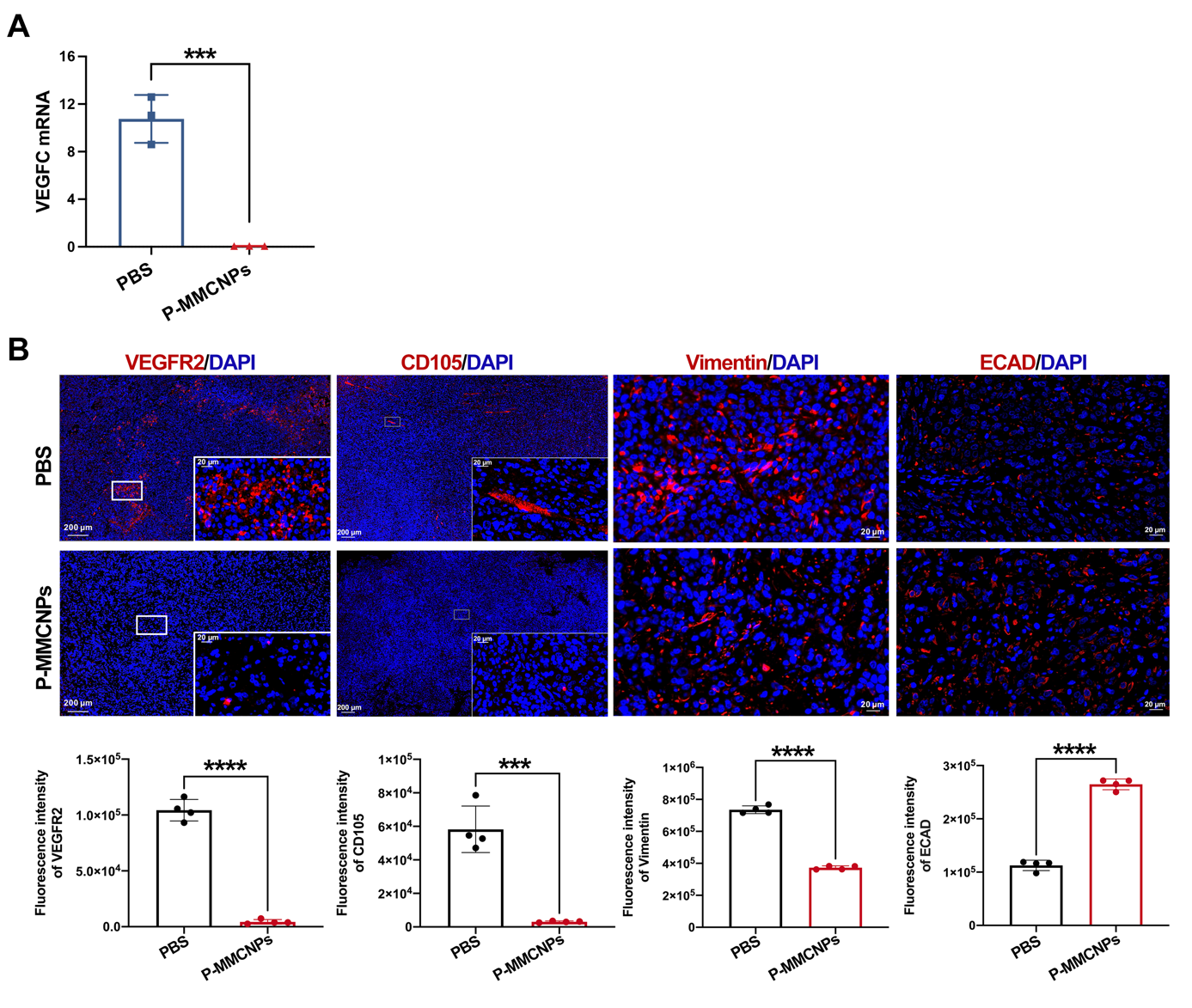


**Figure S21** P-MMCNPs treatment blocks M2 macrophage-related tumor-promoting cascades. (A) VEGFC expression level in resected subcutaneous tumor detected by qRT-PCR. Tumor-bearing mice injected with PBS were used as the control. (B) Fluorescence intensity quantification of VEGFR2, CD105, vimentin, and ECAD in resected subcutaneous tumor. Over-expressed VEGFR2 and CD105 are reliable markers of tumor angiogenesis. ECAD reduction and vimentin increment are regarded as the typical hallmarks of epithelial-mesenchymal transition. Scale bars: 200 μm and 20 μm. Data were presented as mean ± SD from at least three independent experiments. ^***^*P* < 0.001; ^****^*P* < 0.0001.


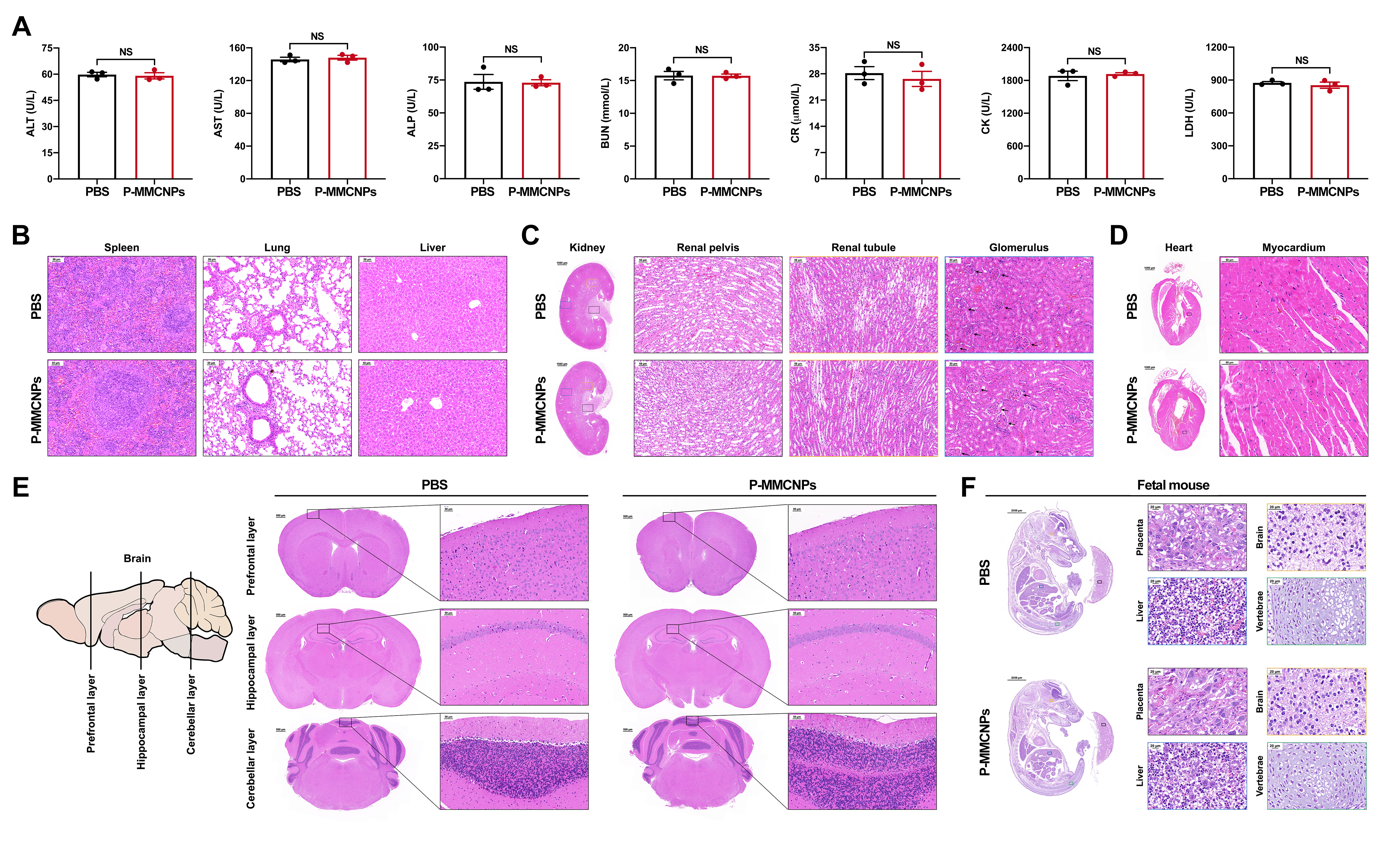


**Figure S22** Organ-toxicity analyses of P-MMCNPs in vivo. (A) Serological detection of liver, kidney, and myocardium. (B-F) Peripheral organ toxicity (B-D), neurotoxicity (E), and embryotoxicity (F) of P-MMCNPs were evaluated by H&E staining. Mice injected with PBS were used as the control. Scale bars: 2000 μm, 1000 μm, 500 μm, 50 μm, and 20 μm. Data were shown as mean ± SD. n = 3 for each group. NS, no significant.

**Table S1** Drug loading property of P-MMCNPs.

| **FeAuNPs (μg)** | **DM1 (μg)** | **PSMA-macrophage membrane (μg)** | **Encapsulation efficiency (%)** | **Drug loading capacity (%)** | **Theoretical drug loading (%)** |
| --- | --- | --- | --- | --- | --- |
| 200 | 2.5 | 200 | 91.46 | 0.91 | 0.57 |
| 200 | 2.5 | 400 | 92.15 | 0.66 | 0.38 |
| 200 | 2.5 | 600 | 92.72 | 0.52 | 0.29 |
| 200 | 5 | 200 | 85.20 | 1.70 | 1.05 |
| 200 | 5 | 400 | 87.94 | 1.23 | 0.73 |
| 200 | 5 | 600 | 88.36 | 0.98 | 0.55 |
| 200 | 10 | 200 | 83.86 | 3.35 | 2.05 |
| 200 | 10 | 400 | 84.22 | 3.21 | 1.38 |
| 200 | 10 | 600 | 86.79 | 1.93 | 1.07 |
| 400 | 10 | 400 | 87.36 | 1.75 | 1.08 |
| 600 | 10 | 400 | 90.89 | 1.40 | 0.90 |
| 800 | 10 | 400 | 91.71% | 1.15 | 0.76 |
